# Wrangling Phosphoproteomic Data to Elucidate Cancer Signaling Pathways

**DOI:** 10.1371/journal.pone.0052884

**Published:** 2013-01-03

**Authors:** Mark L. Grimes, Wan-Jui Lee, Laurens van der Maaten, Paul Shannon

**Affiliations:** 1 Division of Biological Sciences, University of Montana, Missoula, Montana, United States of America; 2 Pattern Recognition and Bioinformatics Group, Delft University of Technology, CD Delft, The Netherlands; 3 Fred Hutchison Cancer Research Institute, Seattle, Washington, United States of America; University Hospital of Modena and Reggio Emilia, Italy

## Abstract

The interpretation of biological data sets is essential for generating hypotheses that guide research, yet modern methods of global analysis challenge our ability to discern meaningful patterns and then convey results in a way that can be easily appreciated. Proteomic data is especially challenging because mass spectrometry detectors often miss peptides in complex samples, resulting in sparsely populated data sets. Using the R programming language and techniques from the field of pattern recognition, we have devised methods to resolve and evaluate clusters of proteins related by their pattern of expression in different samples in proteomic data sets. We examined tyrosine phosphoproteomic data from lung cancer samples. We calculated dissimilarities between the proteins based on Pearson or Spearman correlations and on Euclidean distances, whilst dealing with large amounts of missing data. The dissimilarities were then used as feature vectors in clustering and visualization algorithms. The quality of the clusterings and visualizations were evaluated internally based on the primary data and externally based on gene ontology and protein interaction networks. The results show that t-distributed stochastic neighbor embedding (t-SNE) followed by minimum spanning tree methods groups sparse proteomic data into meaningful clusters more effectively than other methods such as *k*-means and classical multidimensional scaling. Furthermore, our results show that using a combination of Spearman correlation and Euclidean distance as a dissimilarity representation increases the resolution of clusters. Our analyses show that many clusters contain one or more tyrosine kinases and include known effectors as well as proteins with no known interactions. Visualizing these clusters as networks elucidated previously unknown tyrosine kinase signal transduction pathways that drive cancer. Our approach can be applied to other data types, and can be easily adopted because open source software packages are employed.

## Introduction

Cell behavior is controlled by functional interactions among biological molecules, which have been classically studied one at a time, and communicated with pathway diagrams or cartoons. Signaling networks are actually much more complicated than these simple models, as revealed by large-scale approaches to studying the genome, transcriptome, and proteome. These studies produce a large amount of data that are difficult to comprehend *prima facia*. To overcome this problem, a combination of statistical analysis and visualization techniques may be helpful [1]–[4].

A major challenge when dealing with large data sets is how to resolve relationships in the data, and display results in a meaningful way for exploration, presentation, and ultimately, comprehension of the dynamics of cell responses in diseased states and normal differentiation [3]. Much work has been done on exploratory data analysis and inferential statistics [5], and on the “network” metaphor, which describes relationships between biological molecules [6]. Hierarchical clustering dendrograms, heat maps, and network graphs have been employed in attempts to visualize patterns that may indicate functional relationships among different groups within data. It is widely acknowledged that high-throughput characterization technologies will benefit from improved visualization and bioinformatic tools [7], and this is particularly true for phosphoproteomic data analysis [4], [8], [9].

Higher resolution of data structure and computer visualization could be particularly helpful for studies exploring the phosphorylation of cellular proteins. Phosphoproteomic techniques have become increasingly effective in identifying proteins in recent years. Comprehending the resulting data, however, is difficult, both because of the dynamic nature of cell signaling, and because signaling displays many overlaps and great redundancy [10], [11]. To understand these data and transcend limitations imposed by representing signal transduction as linear pathways, there is a clear need for tools and methods that integrate data analysis and graphing [2], [12]. The tools should enable investigators to select statistical techniques with appropriate underlying assumptions for the type of data being analyzed, and visualize results in a way that suggests hypotheses for further data collection and experiments.

One consideration that is especially important when analyzing proteomic mass spectrometry data is how missing values are handled. With careful application of high-resolution instruments, mass spectrometry has a very low false positive rate [13], which means that we may have high confidence in data where proteins are identified. Nonetheless, the false negative rate is likely to be high and in phosphoproteomic analysis is subject to the extent of optimized sample enrichment [14], peptide fractionation [15], [16], phosphorylation site stoichiometry [17] and the mass spectrometer resolution, with recent improvements aiming to minimize the fraction of peptides in complex samples that miss the detector [18]. Most commonly used software tools for statistical analyses, such as *k*-means or hierarchical clustering, require an imputation approach to deal with missing data. Imputing zeros as placeholders to represent the lack of data is a very simple approach that is often used. Imputing zeros is inappropriate for these data, however, because zero values influence the statistical calculations when they are treated as data. Alternative methods to estimate missing values based on previous data have been described, but these methods are suitable when only a few values are missing [19]–[21], or when very strong assumptions can be made on the covariance structure of the data [22], [23] that are unrealistic for proteomic data. It is unreasonable to make inferences about missing values using these methods in phosphoproteomic data because there may be more missing values than data. Therefore, the most direct approach is to calculate statistical relationships using only the observed variables and to ignore all missing variables. We used this approach as a starting point to seek improved methods for resolution of data structure, which we applied to phosphoproteomic data from lung cancer samples [24]. This approach significantly improved the resolution of clusters identified in sparse data sets typical of proteomic studies. Moreover, our analysis of gene function annotations and protein-protein interactions within clusters suggested several novel cancer driver pathways and potential links between these pathways and proteins that have not previously been characterized.

## Results

### Embedding and Clustering Methods

Groups of proteins phosphorylated in the same samples may indicate signaling pathways activated in different classes of tumors, so it is worthwhile to attempt to find clusters defined by statistical methods in phosphoproteomic data. Phosphoproteomic data from Rikova *et al.* [24] were reexamined to elucidate relationships between proteins phosphorylated in lung cancer samples that were not previously appreciated. This dataset, which comprises tyrosine phosphorylated proteins from 41 non-small cell lung cancer (NSCLC) cell lines and over 150 NSCLC tumors, was converted to a table of 2482 genes by 233 samples, is particularly challenging for clustering algorithms because 95.7% of table cells contain no data. Many proteins were identified only in subsets of samples, and we cannot know whether these are truly absent or simply not detected. Use of zeros to represent no data would obscure statistical calculations because all the zeros correlate with each other. Our approach with R software allowed us to explore the use of NA (interpreted as data not available) as a value that was more appropriate than zero to represent the absence of data.

**Figure 1:**
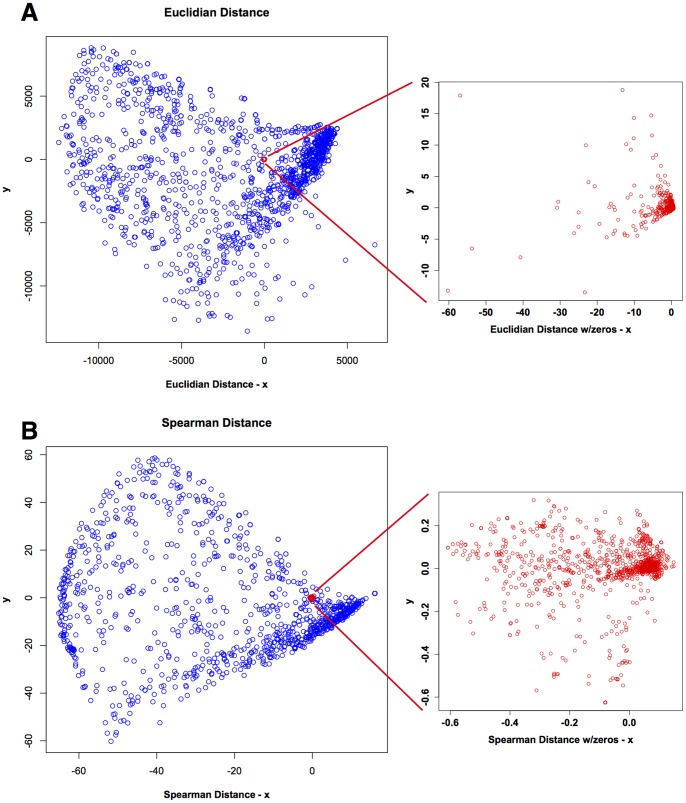
Comparison of two-dimensional Euclidean (**A**) **and Spearman** (**B**) **distance matrices calculated from data where NAs** (**blue points**) **or zeros** (**red points**) **were used to represent the absence of phosphoproteomic mass spectrometry signals.** Data are plotted on the same scale in the main graphs; insets show the scale and distribution of nodes from distance matrices calculated from data using zeros to represent no signals.

We analyzed the data with or without imputing zeros for NAs using two commonly used statistical measures of distance: Pearson or Spearman distance, which is one minus the absolute value of the Pearson or Spearman correlation between each protein and every other protein, and Euclidean distance, which measures the relative closeness in multidimensional space of each protein to every other protein. Pearson and Spearman correlations were very close to one another, so Spearman was used for subsequent analyses. Conversion of the data into statistical distance allows no relationship (a distance of NA) to be set to an arbitrarily large value (100 times maximum real distance between any two proteins; see Materials and Methods). Distance matrices were then converted using multidimensional scaling to Cartesian coordinates in two or three dimensions to visualize data structure (Figures 1 and S1). Using NAs to represent missing values gave rise to data structures (Figure 1, blue points) that were much more highly resolved than those where zeros replaced NAs (Figure 1, red points).

Three-dimensional statistical data structures resolved by Spearman (Figure S1 A, B) and Euclidean (Figure S1 C, D) distance were very different from each other because they employ distinct methods to calculate statistical relationships. Some proteins that were not well resolved by one method were separated by the other, suggesting that a combination of these two methods should further resolve the data. Combining different sources of dissimilarity has been found to be useful in pattern recognition since different dissimilarity measures may emphasize different types of information [25]. The scaled sum of Spearman and Euclidean distance, derived from calculations with NAs to represent the absence of data, was represented as two or three dimensional Spearman-Euclidean Dissimilarity (SED) (Figure S1, E, F; Figure S2, A, B; Movie S1).

### Evaluation of Clustering Methods

We asked whether different clustering algorithms could discern relationships in these data. Graphs of data structure produced by multidimensional scaling, in which node size and color represented the total amount of phosphopeptides, suggested relationships among proteins that could be appreciated by manual exploration of the data structure in Cytoscape (Figure S1). Exploration and selection of clusters based on proximity within the data structure in three dimensions using PyMOL was also possible (Figure S2, Movies S1, S2; see below). Because manual selection of clusters in large data structures is laborious, we evaluated automated selection of clusters using *k*-centers, *k*-means, and multidimensional scaling and t-distributed stochastic neighbor embedding (t-SNE, ref. [26]) using the minimum spanning tree method to select groups based on proximity.

**Figure 2:**
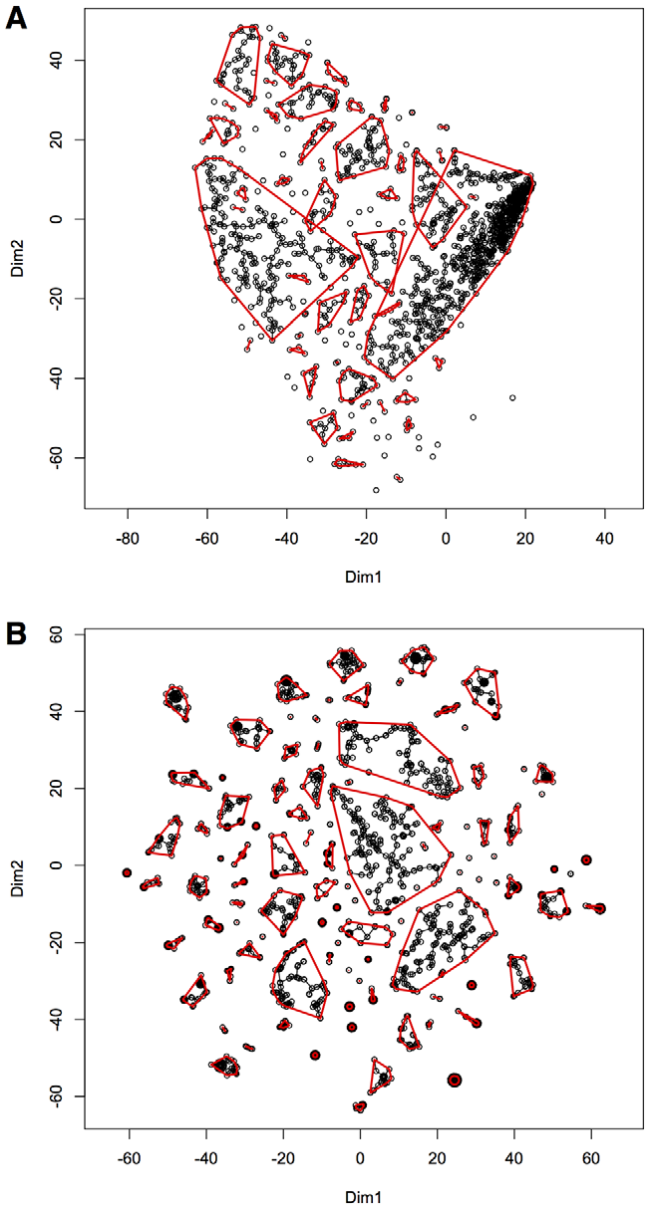
Spearman-Euclidean dissimilarity (**SED**) **reduced to two dimensions by multidimensional scaling** (**A**) **or t-SNE** (**B**)**.** 100 clusters were selected by single linkage minimum spanning trees. Red circles are drawn around the clusters.

To evaluate clusters, an index was calculated from the original data that measured the density of data and number of genes that fitted the overall pattern of expression in each cluster (see Materials and Methods and Table 1).This index ranked clusters containing commonly phosphorylated proteins higher than clusters more sparsely populated with data (higher percent NA, Table 1). Based on this benchmark, the most effective clustering method was the minimal spanning tree method in the t-SNE embedded space. t-SNE is a new pattern recognition technique that aims to model the local structure of the data in a single map whilst ensuring that dissimilar groups of point are modeled far apart [26]. Figure 2 compares clusters identified by minimal spanning tree in multidimensional scaling (A) and t-SNE (B) embedded space from the Spearman-Euclid dissimilarity. (Figure S3 shows two-dimensional t-SNE graphed in Cytoscape; Figure S2C, D and Movies S1, S2 shows three-dimensional t-SNE embedding graphed using PyMOL.) Empirically, we found that t-SNE resolved clusters from the combined Spearman-Euclid dissimilarity more effectively than from either Spearman or Euclid dissimilarity alone (highest sum Index, Table 1). In general, cluster membership defined by different methods increasingly diverged when grouping proteins that were more sparsely represented in the data. Clusters were resolved most effectively when the distance matrix was treated as a “feature vector” in a so-called dissimilarity representation (compare Method: dissimilarity vs. distance, Table 1) [27]. Clustering methods applied to the raw data, or to data where zeros represented the absence of data, were not successful (not shown); they converged on only one large cluster, leaving a number of individual proteins.

**Table 1:** Evaluation of clustering methods.

Method	sum percent NA	sum percent single sample genes	sum percent single gene samples	max Index	sum Index
2D t-SNE Spearman-Euclid dissimilarity	5,524	5,567	7,057	3,455,483	4,716,675
3D t-SNE Spearman-Euclid dissimilarity	5,887	5,275	6,538	3,455,483	4,185,719
*k-*means Euclid dissimilarity	7,401	4,577	5,824	3,737,163	4,055,601
2D t-SNE Spearman dissimilarity	5,797	1,300	6,040	3,506,440	3,986,267
2D t-SNE Pearson dissimilarity	5,616	1,200	5,825	3,506,440	3,957,852
*k-*means Spearman-Euclid dissimilarity	7,129	5,205	5,880	3,484,358	3,814,431
*k-*means Spearman dissimilarity	7,379	300	6,208	3,371,621	3,708,254
2D t-SNE Euclidean dissimilarity	6,536	5,715	6,772	3,094,294	3,234,739
3D t-SNE Euclidean dissimilarity	7,268	5,287	6,295	3,094,294	3,222,101
3D t-SNE Spearman dissimilarity	3,968	4,200	7,202	2,438,734	2,935,132
2D t-SNE Pearson correlation	6,066	400	5,730	405,600	1,064,420
3D t-SNE on 3D MDS of Spearman- Euclid dissimilarity	6,256	7,304	5,895	445,093	939,504
3D t-SNE on 10D MDS of Spearman- Euclid dissimilarity	5,511	7,813	6,178	378,635	881,679
*k*-centres Spearman distance	2,639	6,598	7,814	512,751	805,529
MDS on 3D Spearman-Euclid distance/ dissimilarity	2,067	6,315	8,620	741,469	752,509
MDS on 2D Spearman-Euclid distance/ dissimilarity	3,075	5,683	8,065	322,357	601,140
*k*-centres Spearman-Euclid distance	2,902	7,443	7,805	81,814	347,522
*k*-centres Euclid distance	3,741	2,420	2,761	97,650	253,401

Data ere sorted by sum Index. The Index used for cluster evaluation was defined as.

Index  =  intensity * (1 + realsamples) * (1 + cleargenes)/(1 + percent NA).

Where

intensity  =  total signal – (total signal * percent NA/100).

cleargenes  =  no. genes – genes culled by slope.

realsamples  =  no. samples – (no.samples * percent single gene samples/100).

Single gene samples is the number of cases where a sample in the cluster contains only one gene. Single sample genes is the number of cases where a gene in the cluster is represented in only one sample. The “culled by slope” function sorts genes and samples from largest to smallest within each cluster and measures the slope of the regression line for each gene in all the samples. If the slope is negative, the gene follows the general pattern in the cluster. If the slope is positive, the gene is more highly expressed in different samples than the rest of the group, and is culled. Data are sorted by sum Index, which is the sum of all Index values from 100 clusters.

100 clusters were resolved by each method for comparison. MDS  =  multidimensional scaling. t-SNE  =  t-distributed stochastic neighbor embedding. t-SNE was not effective when attempting to preserve distance from distance matrices (not shown), but it was very effective when treating data as a feature vector representation of dissimilarity. t-SNE was used to create maps reduced to 2 or 3 dimensions. Minimum spanning tree, single linkage method was used to resolve clusters from MDS and t-SNE.

26–30 clusters were identified from fuzzy *c-*means scores by selecting membership by scores greater than the mean score plus 2.5 times the standard deviation (not shown). All but 11 of these clusters were similar, containing 100–140 of the most highly represented proteins in the data set with a mean overlap of about 40 proteins. Only 200–232 of 2482 genes were grouped into clusters by this method.

### Data Wrangling

The concept of “fuzzy clustering” embraces the notion that membership in more than one group is possible. Unfortunately, fuzzy *c-*means clustering resolved only a few distinct clusters containing less than 10% of the proteins in the data set (see Table 1 legend). Though this particular clustering technique proved to be of limited use for these data, the concept of fuzzy or overlapping boundaries between clusters is nevertheless important to keep in mind when examining clusters determined by any method. Membership in individual clusters identified from hard clustering methods on Spearman, Euclidean, or SED embedding split in different ways clusters that contain even the most statistically well-represented proteins (Figure S4). We seek to appreciate patterns of tyrosine phosphorylation to elucidate different pathways that may drive or be active in different types of lung cancer. While it is worthwhile to carefully determine which sets of proteins are most often co-activated, tyrosine phosphorylated proteins found in many samples may be activated by multiple overlapping pathways, and one or more downstream effectors may be activated by more than one tyrosine kinase [28], [29]. Thus, assignment of proteins to one cluster should not be viewed as evidence for excluding it from participating in a signaling pathway identified in another cluster.

With this in mind, we investigated how data-driven analyses coupled with hypothesis-driven interrogation and filtering may be used to glean more information from the lung cancer data set. We hypothesized that the presence of one or more tyrosine kinases in individual clusters implicates those kinases in pathways (whether direct or indirect) that cause tyrosine phosphorylation of other proteins in that cluster. Thus, we provisionally identified clusters by tyrosine kinases, where present. Clusters that contained the most highly phosphorylated proteins in these data contained FAK (PTK2), LCK, LYN, FYN, DDR1 and EGFR. We focussed on these clusters, and two other clusters containing ALK and MET, for detailed investigation. We evaluated and filtered clusters based on internal criteria, that is, based on the primary data, and external criteria from protein interaction and gene ontology (GO) databases [30]–[32].

To evaluate the validity of clusters, we examined the subset of the primary data contained within them. We focused on the clustering methods that performed well according to the criteria defined in Table 1. Data were graphed as heat maps sorted by descending phosphopeptide contents. The sorted heat map, which can be considered a three-dimensional histogram with the *z*-dimension representing quantity by a color scale, provides an overview to evaluate conformity to a similar pattern in the primary data. Clusters containing the most highly represented proteins in the lung cancer data are shown in Figures S4 and S5. Clusters were also evaluated using the index that measures data density as described above (Table 2). FAK (PTK2) and LCK were grouped together with MAPK14 (p38α) and GSK3A (which was present in all samples) by all measures except Spearman (Figure S4C; Table 2, Spearman t-SNE group 108). Clusters containing EGFR were also largely similar, grouping EGFR with DDR1, LYN, and FYN (Figure S5), except that *k-*means on Euclidean embedding grouped EGFR with the FAK-LCK cluster (Figure S4A; Table 2, Euclid *k*-means group 56). Despite these exceptions, there was significant agreement among different clustering methods for the most highly represented proteins in the data set.

**Table 2:** Summary of key clusters.

Kinase	Method	Group	Rank	no. genes	percent single sample genes	no. samples	percent single gene samples	total signal	percent NA	Index
FAK(PTK2)	Spearman t-SNE	51	1	14	0	233	0	6,340	5	3,506,440
FAK(PTK2)	SED t-SNE	71	1	18	0	233	0	7,086	7	3,455,483
FAK(PTK2)	Combined Filtered	NA	NA	13	0	233	0	6,346	8	2,077,545
FAK(PTK2)	Euclid k-means	56	1	30	0	233	0	9,572	14	3,737,163
LCK	Spearman t-SNE	108	3	8	0	232	4	1,291	28	64,074
FAK(PTK2)	Euclid t-SNE	37	1	42	0	233	0	9,548	33	1,932,687
EGFR	Combined Filtered	NA	NA	12	0	232	0	2,770	32	175,211
EGFR	Spearman t-SNE	22	2	22	0	233	1	3,266	38	279,034
EGFR	SED t-SNE	14	2	33	0	233	0	4,566	42	465,278
DDR1	Euclid k-means	9	2	23	0	232	2	3,041	43	205,086
EGFR	Euclid t-SNE	14	2	23	0	231	0	3,132	44	209,573
MET	Combined Filtered	NA	NA	30	0	46	0	919	42	17,714
MET	Spearman t-SNE	76	7	8	0	161	24	419	56	3,627
MET	Euclid k-means	17	5	23	0	213	17	890	74	9,332
MET	SED t-SNE	40	5	14	0	168	36	386	77	1,630
MET	Euclid t-SNE	12	3	162	0	229	3	3,207	86	120,157
ALK	Combined Filtered	NA	NA	26	0	9	0	79	62	78
ALK	Spearman t-SNE	44	43	9	0	48	71	61	79	24
ALK	Euclid k-means	54	18	18	0	103	50	92	88	92
ALK	Euclid t-SNE	52	19	23	35	83	63	79	91	35
ALK	SED t-SNE	28	12	175	62	155	40	264	98	229
EML4	SED t-SNE	121	19	4	0	73	67	56	63	40
EML4	Euclid k-means	55	19	20	0	103	59	93	90	82
EML4	Euclid t-SNE	40	25	55	42	75	65	77	96	12

Data were sorted by increasing percent NA for each group, identified by the most well-represented tyrosine kinase in the cluster, except for EML4†, which is not a kinase, but shown here because it was found to be linked in a chromosomal translocation to the tyrosine kinase domain of ALK [24]. Clusters were evaluated as described in Table 1 and Materials and Methods. Those identified by automated techniques using dissimilarity as a feature vector are labelled. Clusters determined by combining and filtering are identified as “Combined Filtered.” Rank refers to the rank by Index comparing groups from that particular method; Group is the identifier number.

**Figure 3:**
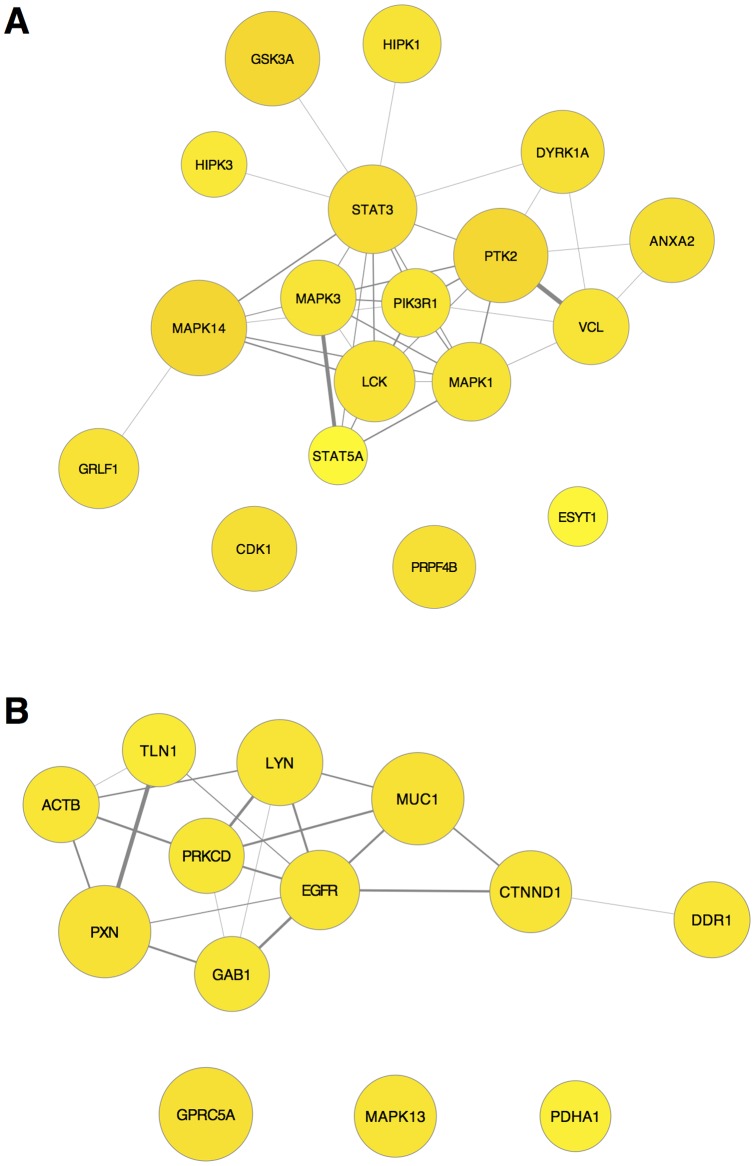
Networks from clusters containing the most highly tyrosine phosphorylated proteins in lung cancer samples. **A**) Cluster containing LCK and FAK (PTK2) derived from t-SNE on SED embedding (Figure S4D). **B**) Cluster containing EGFR and LYN, derived from first performing t-SNE Spearman and Euclidean embedding separately, then combining these clusters and filtering (Figure S5E). Node size and color (white to yellow) indicates the total number of phosphopeptides detected in all samples. Edges are protein interaction data from String (string.embl.de/), GeneMANIA (genemania.org/), and the kinase-substrate data from PhosphoSitePlus (phosphosite.org). For clarity, since graphs of these clusters including all individual edges were difficult to interpret, edges were merged, and edge weights, which indicate the strength of evidence for interaction, were summed to determine the thickness of the edge line. Protein interaction network data was imported into R for the edge merge and plotted with RCytoscape as described in Materials and Methods. Node position in network graphs was set using an edge-weighted, spring-embedded layout in which highly connected nodes group closer together. The cluster in (A) had 107-fold more edges, 544-fold greater edge weight, and 7.5-fold more GO terms retrieved than the average random cluster. The cluster in (B) had 88-fold more edges, 499-fold greater edge weight, and 10.8-fold more GO terms retrieved than the average random cluster. As an additional measure, the number of edges expected from these nodes in the entire lung cancer network was calculated (see Materials and Methods). The LCK/PTK2 network (A) had 122 more edges, and the EGFR network (B) had 67 more edges, than expected by this calculation.

Different embedding (Spearman vs. Euclidean) produced overlapping but distinct clusters, and the combined (SED) embedding produced a reasonable consensus view (Figure S4D, S5D). Considering that both Spearman and Euclidean dissimilarity define clusters that are statistically meaningful, we also combined them in a different way, by merging overlapping groups after clustering, then filtering. Applying this approach to the FAK-LCK group (Figure S4E) returns a cluster very similar to the SED cluster (Figure S4D). Similarly, there was good agreement comparing the EGFR cluster when Spearman and Euclidean embedding was combined before (Figure S5D, SED t-SNE) or after (Figure S5E) the clustering algorithm was performed. These results suggested that combining Spearman and Euclidean embeddings either before or after clustering is useful to represent a consensus view of clusters. The SED (t-SNE) FAK (PTK2) cluster (Figure S4D) and the combined Spearman and Euclidean EGFR cluster (Figure S5E) were graphed as networks in Figure 3, incorporating data from protein interaction databases as edges (explained in External Evaluations, below).

**Figure 4:**
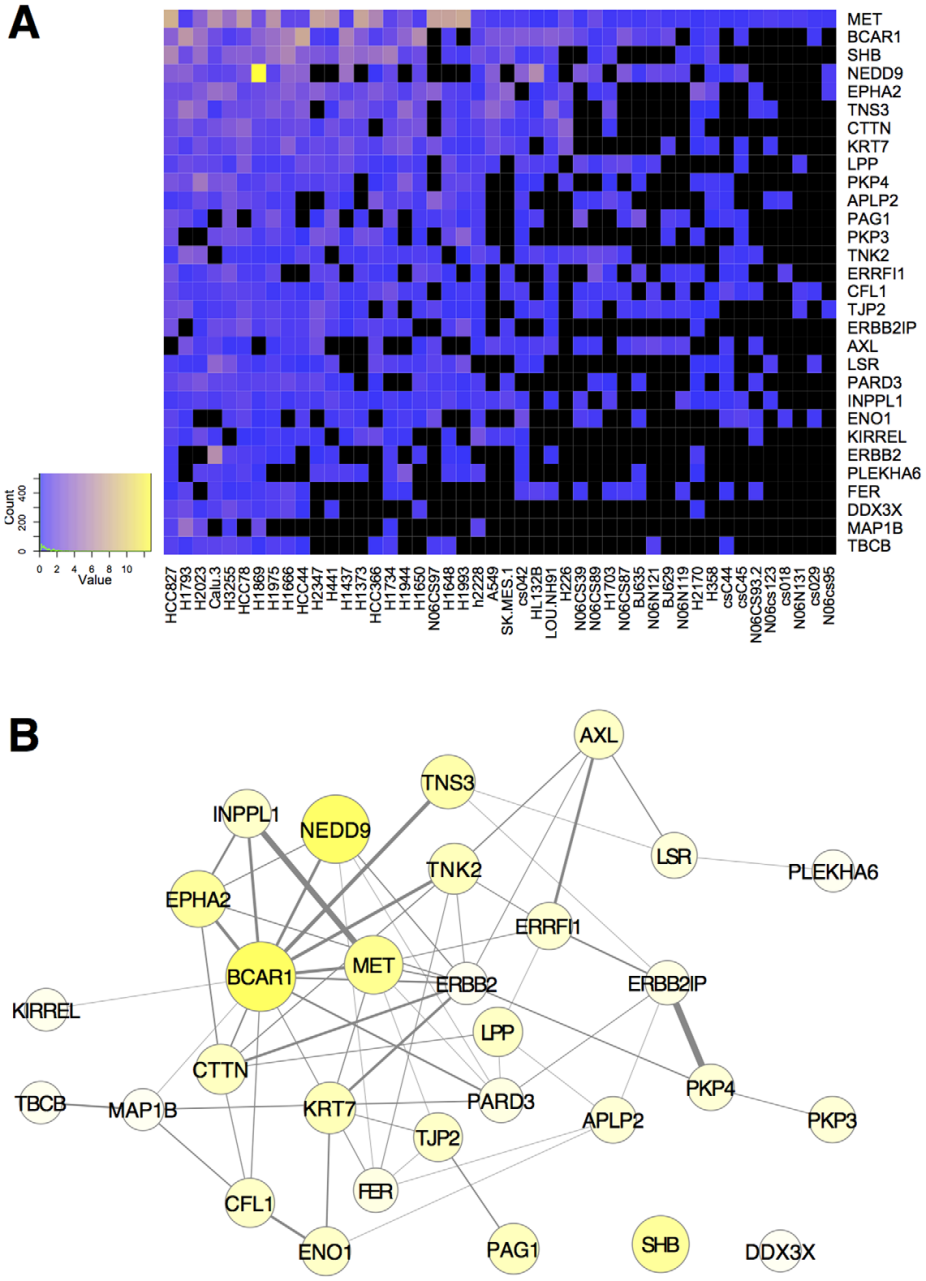
Filtered cluster containing MET derived from first performing t-SNE Spearman and Euclidean embedding separately, then combining these clusters and filtering for samples containing MET and the most highly represented proteins that are consistent with data in the rest of the cluster (**see Materials and Methods**)**.** The heat map (**A**) represents missing data (NA) as black, and increasing scaled peptide counts are shown on a blue-yellow scale (color key, left). Data are ordered by decreasing sums of scaled peptide counts for genes (decreasing from top to bottom) and samples (decreasing from left to right). **B**) MET in lung cancer shown as a protein-interaction network graphed as in Figure 3. This cluster had 70-fold more edges, 847-fold greater edge weight, five-fold more GO terms retrieved than the average random cluster, and 249 more edges than would be expected from these nodes from the entire lung cancer network.

One important goal of detailed analysis of large data sets is to uncover new mechanisms or signaling pathways. MET, the receptor tyrosine kinase for hepatocyte growth factor (HGF) has been shown to drive tumorigenesis when overactivated in a number of cancers, including lung cancer [33]. Anaplastic lymphoma kinase (ALK) is an important oncogenic driver, yet is less well studied than many other receptor tyrosine kinases (RTKs) [34]. Cluster membership for clusters identified from the data examined here containing MET and ALK were more varied when different methods were used (Figures S6,S7,S8, Table 2). Clusters containing MET ranged in size from 8 to 162 proteins, with little overlap (Table 2, Figure S6). None of the clusters identified automatically appeared to be particularly compelling based on internal evaluations, however, combining clusters from t-SNE on Euclidean (Figure S6B) and Spearman (Figure S6C) embedding, then filtering, defined a reasonably-sized cluster that made the most sense by internal evaluations (Figure 4, low percent NA, Table 2). This cluster identified collaboration of the RTKs EPHA2, ERBB2, and ERBB3 with MET, which may provide additional targets for metastatic lung tumors.

Phosphorylated ALK was detected in a smaller number of samples in the data set examined, which creates a difficult statistical problem that requires a combination of approaches to yield potential biological insight. The *k-*means cluster didn't contain proteins whose pattern of phosphorylation in the primary data was well correlated (Figure S7A), and the SED (t-SNE) cluster containing ALK was very large, containing a number of sparsely-identified proteins (Figure S7D). The only genes with similar cluster patterns between t-SNE Euclid and Spearman clusters were ALK and EML1 (Figure S7B, C). We therefore experimented with different approaches to combine and filter clusters.

**Figure 5:**
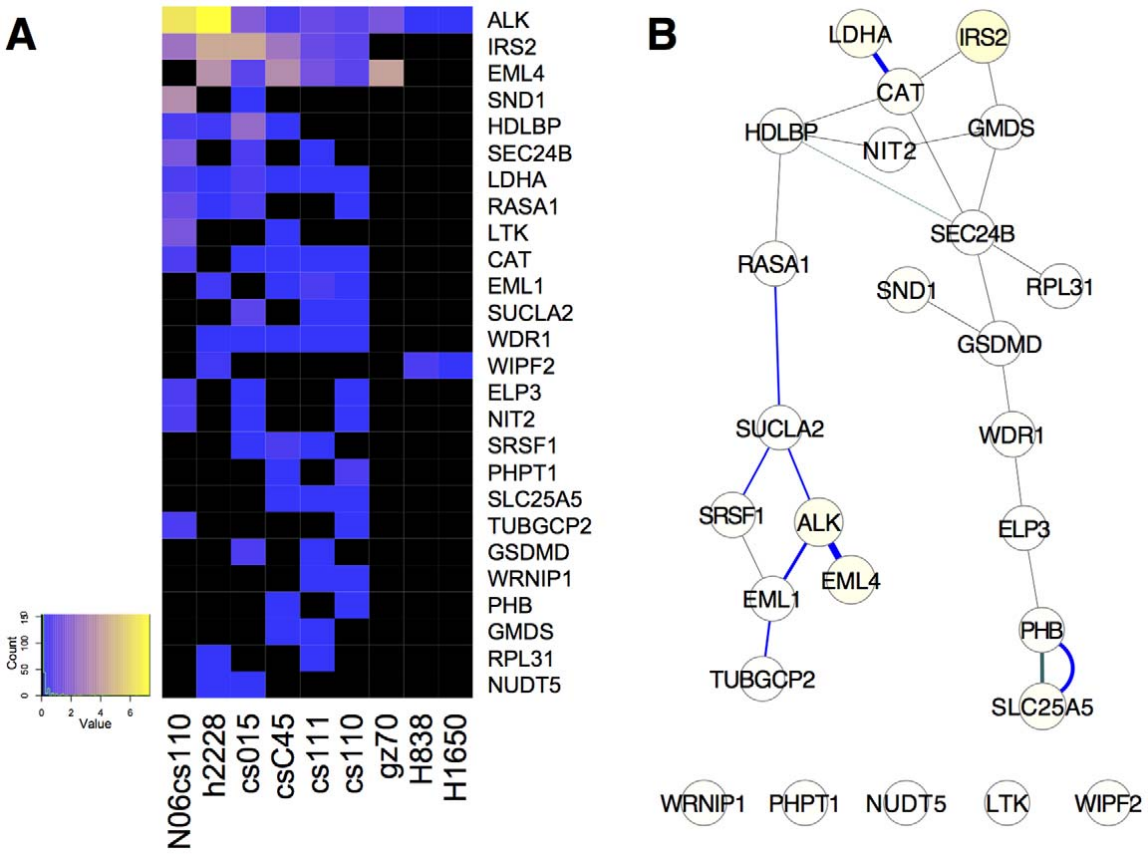
Filtered cluster containing ALK, graphed as a heat map (**A**) **and protein-interaction network** (**B**)**.** This cluster is derived from clusters combined from Figure S8B and C in which proteins present in a single sample, or samples containing a single gene, were filtered. This cluster had twelve-fold more edges, ten-fold greater edge weight than the average random cluster, and 7 more edges than would be expected from these nodes in the entire lung cancer network. Individual edges are shown from String (blue) and GeneMANIA (black).

ALK and Echinoderm microtubule associated protein like 4 (EML4) were correlated in 6 samples, which was identified in the Spearman (t-SNE) cluster (Figure S7C). This was noted by Rikova, *et al.,* who elegantly proved that a chromosomal translocation produced a hybrid *ALK-EML4* gene in a subset of cases, creating an oncogene analogous to nucleophosmin-anaplastic lymphoma kinase (NPM-ALK), which drives anaplastic large-cell lymphomas [24], [34], [35]. There are more cases, however, where EML4 was detected and ALK was not (Figure S8A), and cases where ALK was detected and EML4 was not (Figure S8B). In addition, there are a number of proteins identified in one sample that contains EML4 but not ALK (H3255, Figure S8A, B). These data affected Euclidean dissimilarity more than Spearman, and thus mask potentially interesting relationships. A more informative clustering was produced by first combining clusters from different methods (Figure S8C), and then filtering for ALK and proteins present at least twice (Figure 5).

Because the methods to identify ALK and MET clusters (Figures 4 and 5) involved several steps beyond clustering algorithms, that is, combining clusters and filtering in various ways, we describe these methods as “data wrangling.” This term is intended to denote some curating of the data into groups using quantitative filters, starting with clusters identified by automatic methods. To further validate these methods, we examined clusters using external evaluations.

### External evaluations

Clusters identified from statistics containing proteins that physically interact are likely to represent functional signaling networks. Protein interaction and GO data retrieved from external databases were used as additional measures of the biological significance and validity of clusters identified above. These databases are incomplete works in progress [36], [37], nevertheless if the clusters implicate real pathways they will be more likely than a random selection of genes from the dataset to show interactions and functional synergy. As a control, we randomly selected 11 to 34 proteins from the dataset (the size of clusters we deemed informative) and determined the average number and weight of edges that represent evidence for physical or genetic interactions for random clusters (see Materials and Methods). The networks shown in Figures 3 and 4B all had more than sixty-fold more edges (and 500-fold more edge weight) over background from randomly selected proteins (see Figures 3 and 4 legends).

We used random clusters to determine the background GO term enrichment, which was about one enriched GO term for every three genes selected randomly from the lung cancer data set (see Materials and Methods). This relatively high background for GO term enrichment indicates that GO terms for the clusters should be interpreted with caution. Nonetheless, the number of GO terms retrieved were more than five-fold over background for FAK (PTK2), EGFR, and MET networks (Figures 3 and 4). A summary of GO terms for these clusters, and all clusters identified by t-SNE on SED 2D embedding (cluster membership and GO summary tables, available online), revealed links to many signaling, metabolic, and growth-control process in the FAK (PTK2) group, implicating these proteins as hubs of signal integration for many lung cancer signaling pathways. The EGFR cluster also had links to signal transduction and growth control, and also to differentiation. In contrast, the MET cluster had many more links to cell migration, control of actin organization, and adhesion, suggesting a role for these proteins in metastasis.

Proteins in the ALK cluster are not as well-studied, and the ALK cluster GO terms were not significantly increased over background, yet eleven-fold more edges (and ten-fold more edge weight) were present in the ALK network compared to random proteins (Figure 5). The observation that eleven-fold more edges (and ten-fold more edge weight) were present in the ALK network compared to random proteins indicated that the ALK cluster is worthy of further investigation.

### Co-activation of tyrosine kinases in lung cancer

**Figure 6:**
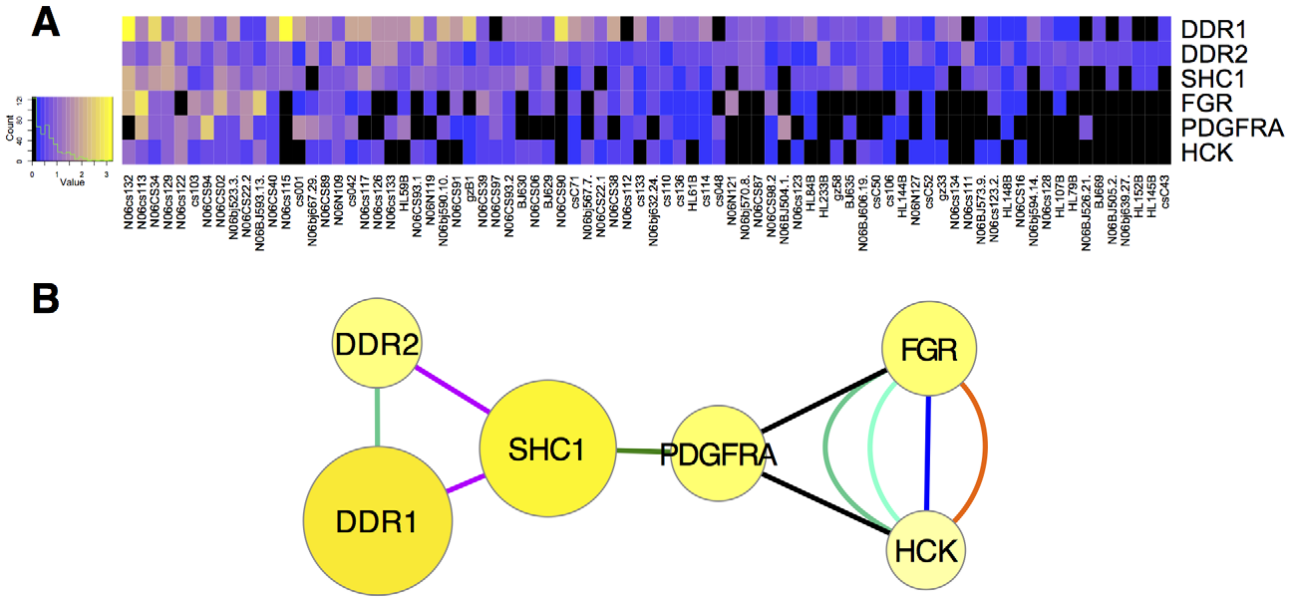
Filtered cluster containing DDR2. (**A**), graphed as a heat map; and (**B**), graphed as a network as in Figure 5, except additional edges are included from GeneMANIA: black – genetic interactions; dark turquoise – shared protein domains; violet – physical interactions; green – pathway; and String: light turquoise – homology; orange – knowledge; and blue – combined score. SHC1 was included because it connected the network for these proteins for which limited interaction data is known.

31 of the 58 RTKs in the human genome were detected in this dataset, and all nine SFKs. The co-activation of RTKs and SFKs observed in clusters containing EGFR (Figure 3B) and MET (Figure 4) suggested the hypothesis that functional synergy between two or more tyrosine kinases plays a role in lung cancer development. This prompted us to search for other clusters in which two or more tyrosine kinases were found together. We identified clusters defined from t-SNE embedding of Spearman, Euclidean, or combined (SED) dissimilarity as described above that contain two or more tyrosine kinases (Table 3). Discoidin domain receptor 2 (DDR2) has recently been identified as a possible lung cancer driver [38], and was associated with the SFK, HCK in clusters derived from all three of these embeddings (Table 3). DDR2 was frequently co-activated with HCK, and also with DDR1, FGR, and PDGFRA in a number of samples, as identified in the SED cluster (Figure 6). These clusters of co-activated tyrosine kinases indicate cooperation in signal transduction, and may suggest therapies with combinations of kinase inhibitors [39], [40].

**Table 3:** Tyrosine kinases in clusters.

Gene Name	Spearman cluster	Gene Name	Euclid cluster	Gene Name	SED cluster
EGFR	22	DDR2	37	AXL	14
FYN	22	FGR	37	EGFR	14
LYN	22	HCK	37	FYN	14
DDR2	90	LCK	37	LYN	14
HCK	90	PDGFRA	37	DDR1	37
EPHA4	63	DDR1	14	DDR2	37
TYRO3	63	EGFR	14	FGR	37
EPHA3	23	FYN	14	HCK	37
YES1	23	LYN	14	PDGFRA	37
FGR	41	AXL	12	EPHA2	12
PDGFRA	41	EPHA1	12	EPHA4	12
CSF1R	37	EPHA2	12	EPHB2	12
KIT	37	EPHA3	12	ERBB3	12
SRC	37	EPHA4	12	ROR1	12
EPHA5	32	EPHB2	12	TYRO3	12
ERBB4	32	EPHB3	12	EPHA1	58
FGFR4	32	ERBB2	12	EPHA3	58
		INSR	12	EPHB3	58
		MET	12	ERBB2	40
		ROR1	12	INSR	40
		TYRO3	12	MET	40
		YES1	12	FRK	119
		BLK	5	YES1	119
		SRC	5	NTRK1	11
		EPHA5	13	SRC	11
		ERBB4	13	ALK	28
				EPHA5	28
				ERBB4	28
				LTK	28

Clusters from Spearman, Euclid, or SED dissimilarity and t-SNE were filtered for the presence of two or more tyrosine kinases. Numbers identify the particular cluster from each embedding method.

## Discussion

This paper addresses urgent calls to analyze proteomic data with more effective methods, and integrate these analyses with protein interaction and function databases to elucidate signaling networks that drive diseases such as lung cancer [41], [42]. Combining data interrogation methods with computer visualization tools significantly augments our capacity to make sense of large data sets and their links to genome and protein interaction databases. We describe here effective approaches to explore data structure, select subsets based on statistical relationships, and visualize selections as networks. The combined internal and external evaluations provided strong evidence that clusters of proteins identified here represent functional signaling networks in lung cancer because they contain proteins that are known to interact with each other.

The open-source software platforms R, Cytoscape, and RCytoscape were employed for this study. Scripting languages such as R are much more adept at handling large data sets than spreadsheets, and R has a rich library of statistical analysis tools, including many developed for bioinformatics and systems biology [1], [43]. Cytoscape is arguably the most advanced tool for network graphing, and offers a graphic user interface (GUI) well suited for exploration and analysis of networks [44], [45]. RCytoscape (rcytoscape.systemsbiology.net) links R and Cytoscape, and extends Cytoscape's functionality beyond what is possible with the Cytoscape GUI.

Key steps that resolved informative clusters were: **1**) Calculation of distance matrices using NA to represent the absence of data proved appropriate for mass spectrometry-based proteomic data, and would be advantageous for any data set where detection limits significantly compromise confidence about negative results. **2**) Dissimilarity matrices were used as feature vectors for embedding. Embedding dissimilarity representation may resolve data structure more effectively than the distance matrix because no attempt to preserve distance is made [27]. **3**) Multiple methods were used for statistical calculation of dissimilarity. A combination of Spearman (or Pearson) and Euclidean distance may increase the resolution of the statistical data structure [25], or clusters identified by different methods may be combined later. **4**) t-SNE was employed for embedding [26]. We found that t-SNE was as good or better at resolving clusters from proteins well-represented in the data than other methods, and far superior for identifying clusters from less-well-represented proteins. To explore data structure, displaying three dimensional data structures in PyMOL offered the advantage that the investigator may explore the graph and select clusters of nodes for further analysis (Figure S2, movie S2). Displaying two-dimensional data structure in Cytoscape had the advantage that individual node names were visible (Figures S1, S3). **5**) Data wrangling was performed where necessary to combine and filter clusters by conformity to a pattern in the primary data, membership, and/or signal strength. Inspection of the clusters' primary data (*e.g.,* using heat maps) was crucial at this stage. This step is termed wrangling because manual, hypothesis-driven manipulation, and decisions based on the results, are akin to herding data into clusters. **6**) Clusters were analyzed using external databases containing protein interaction data and GO terms. **7**) Finally, clusters were visualized as networks to convey a large amount of information in a single graph. Merging edges was useful for clarity where graphs have a large number of edges. String and GeneMANIA use different methods to calculate edge weights, but the weights are of similar scale, so merging them is an acceptable way to provide an overview of evidence for interactions.

This kind of data analysis is an example of pattern recognition for which human brains can be very adept [46], whereas computers are functionally more capable of recognizing patterns in large matrices of numbers. Computer algorithms that embed statistical relationships into two- or three-dimensional structures are thus a valuable first step. We found that automated clustering methods were fairly effective for statistically robust data (Figures S4, S5, and 3), but for more difficult clusters, automated methods were less reliable (Figures S6, S7), so it was advantageous to employ the capabilities of the human brain aided by computer graphics.

The human mind's appreciation of shape also comes into play when constructing informative graphics [47], [48]. Networks of clusters with protein-interaction edges convey the amount of phosphorylation and known interactions in a meaningful way, which is much more informative than grids of colored squares adorned with dendrogram trees. Large, complex network graphs can be useful for computer-aided exploration, but rapidly become unwieldy due to their complexity. Simplification of protein interaction edges and filtering nodes made graphs more accessible (Figures 3,4,5).

### Biological insights

Individual cancerous tumors typically express different combinations of active tyrosine kinases, including multiple receptor tyrosine kinases [24], which makes it difficult to sort out relationships between signaling pathways for targeted therapy. These analyses provide new insights into mechanisms whereby different combinations of tyrosine kinases may delineate distinct divisions of labor that induce cell proliferation, avoidance of apoptosis, and in many cases, promote metastasis. The data-driven clusters suggest potential links between several different cancer driver RTKs, SRC-family kinases (SFKs), RTK-SFK pairs, and proteins that have not previously been characterized.

GO terms enriched in clusters were not randomly distributed, rather there were themes that suggest roles in cell proliferation, differentiation, adhesion and migration, as well as strong links to different metabolic processes such as nucleic acid or carbohydrate biosynthesis, RNA processing, DNA replication, and chromatin structure (GO Summary Tables, Information S1). That different groups were associated with different biological processes further validates the clustering technique, and suggests that proteins were activated by distinct pathways or processes in different tumor samples. While a detailed examination of all the clusters identified from these data was beyond the scope of this paper, the cluster membership and GO summary tables provide a starting point for further investigation. Identification of these new clusters provides a rich source of information to formulate hypotheses for further experiments and predict more effective therapies involving combinations of drugs [39].

Many RTKs shown to be tyrosine phosphorylated in this data set have been identified by other studies to be activated by different mechanisms, for example, INSR; MET; EHPA2; PDGFRA/B, FGFR1, and ALK [39], [49], [50]. The presence of LCK and LYN in clusters containing proteins commonly phosphorylated in lung cancer suggest potential pathways of signal transduction (Figure 3). These are of particular interest in light of studies that justify the use of SFK inhibitors, or a combination of SFK and RTK inhibitors, to treat lung cancer [39], [40]. SFKs associate with RTKs, play a role in transducing their signals, and can phosphorylate RTKs directly, in some cases mimicking those sites phosphorylated during ligand-induced receptor activation [51], [52].

The results shown in Figure 4 expand the list of RTKs that potentially collaborate with MET in lung cancer to include EPHA2, ERBB2 (HER2), ERBB3 (HER3), and AXL. MET amplification in lung cancer has recently been shown to be associated with activation of EGFR, ERBB2, ERBB3, and RET [53]. Co-immunoprecipitation of these RTKs with MET suggests that trans-activation of RTKs can occur through hetero-dimerization [53]. Recently the RTK, AXL has been found to have a key role in determining lung cancer chemosensitivity [54], [55]. Tyrosine phosphorylation of AXL was detected concomitant with that of MET, ERBB2, and EPHA2 in a number of samples, indicated by the cluster shown in Figure 4.

DDR1, which was itself highly tyrosine phosphorylated in the data analyzed here, clustered with EGFR and LYN (Figure 3B). DDR1 was unknown as a cancer driver at the time the Rikova *et al.,* [24] was published; yet this RTK is now known to be a cancer driver that promotes cell survival through Notch1 [56]. Recently, DDR2 has been shown to exhibit elevated mRNA levels in NSCLC samples [38]. Co-activation of MET, AXL, ERBB2, and EPHA2 (Figure 4), and co-activation of DDR1 with EGFR (Figure 3B), DDR2, HCK, PDGFRA, and FGR (Figure 6) is evidence that simultaneous activation of multiple tyrosine kinases may be common in lung cancer. The frequency in which tyrosine phosphorylated driver kinases are detected may suggest priorities for therapies that employ combinations of specific kinase inhibitors, as well as new avenues for research and drug development. Thus, assays for activation of sets of particular kinases in individual tumors may be broadly applicable for indicating appropriate drugs for cancer therapy in the lung and other tissues [57].

A major challenge for both basic research and cancer therapy is to identify critical signal transduction pathways governing cell fate decisions for specific cell types. The clusters identified here from lung cancer phosphoproteomic data, combined with network and GO analysis, suggests that RTK and SFK pathways have some degree of compartmentalization and functional specialization, and will hopefully guide further research and investment of resources to develop drugs targeted to specific proteins or pathways for cancer therapy.

The novel approaches for clustering sparse phosphoproteomic data described here can enhance resolution of complex data sets, which is an important step towards comprehension of molecular signaling networks in cancer. Our results are consistent with those of Naegle, *et al.,* [4], who showed that no single clustering algorithm is sufficient to produce results with biological meaning, and therefore combining and filtering, or wrangling data, and employing external information such as that from protein-protein interaction and GO databases, are crucial for elucidating interesting relationships in the data.

## Materials and Methods

R commands and functions that were used for processing and graphing data are available in Rcommands S1 and Rfunctions S1.

### Phosphoproteomic data

The phosphopeptide data set from Rikova et al. (“20070918_spectrumtable.txt”) was downloaded from PhosphoSitePlus (http://www.phosphosite.org/suppData/RikovaCell/20070918_spectrumtable.xls) [58]. Gene names were mapped to HUGO gene names (http://www.genenames.org/) using the R library “org.Hs.eg.db” and checked against UNIPROT and ENTREZ IDs. All peptide counts for all proteins were summed for each protein in each lung cancer sample. For graphing, “total phosphorylation” represents the sum of phosphopeptides detected for that protein in the entire data set.

### Clustering Methods

The matrix of proteins (gene names) and samples, in which the absence of data is represented by NA, was used to calculate Pearson or Spearman correlations between pairwise complete observations. (We compared this to a simple imputation approach in which zeros were used to replace NA.) We defined a Pearson or Spearman distance as one minus the absolute value of the correlation. Euclidean distance was calculated using the R function, dist. (Calculation of distance using Manhattan or Canberra distances were not appreciably different from Euclidean.) Pearson and Spearman correlations were very similar, and Spearman correlations were used preferably in subsequent steps because these data can't be assumed to be linear.

Spearman and Euclidean data structures had different regions of high and low resolution. In other words, some sets of genes that were poorly resolved in one could be resolved by the other. We combined Spearman and Euclidean distance matrices by first scaling the distance matrices to the same scale relative to one another, and then averaging them, giving rise to Spearman-Euclidean Distance (SED). The SED was treated as a dissimilarity representation [27].

Clustering methods applied directly to distance matrices described above (either with NA or zero to impute the absence of data) were not effective (see Results), so the following procedure was performed. Distances of NA signify no statistical interaction between proteins in these data and thus should be considered large compared to actual distances. These were therefore set to two orders of magnitude higher than the maximum distance in each distance matrix. (Setting this value larger than this had no effect on the data structure.) The resulting distance matrices were used directly, or they were used in a dissimilarity representation [59], i.e. as “feature vectors,” for clustering algorithms (*k*-means [60], *k*-centers [61], fuzzy *c*-means [62]) or dimension reduction techniques (multidimensional scaling, t-distributed stochastic neighbor embedding). A minimum spanning tree method [63] that finds groups that can be connected by a single linkage was used to resolve clusters from MDS and t-SNE using the R functions “distconnected” and “spantree” from package “vegan”.

Multidimensional scaling was performed using the R function, “cmdscale.” Clustering methods fuzzy c-means, *k*-means, and *k*-centers were performed in MatLab using the “kmeans” function in statistics toolbox, “kcentres” function in PRtools (prtools.org) and “fcm” function in fuzzy logic toolbox, respectively, by setting the number of clusters to 100. For *k*-means and fuzzy *c*-means, the distance matrices are treated as dissimilarity representation and used as feature vectors; *k*-centers performed the clustering by considering the original distance and using the distance matrices directly.

The R implementation of t-SNE was employed with the following parameters: k = 2 or 3, initial dimensions  = 30, perplexity  = 30, max iterations  = 1000, min cost  = 0, and whiten  =  TRUE. Figure S3 shows the t-SNE graph with node names and total phosphopeptides graphed, where grouping of highly phosphorylated proteins is apparent. One advantage of t-SNE is that the number of clusters does not have to be determined in advance; it can be determined by close proximity on the t-SNE map. t-SNE measures the similarity between points *a* and *b* by centering a bivariate Student-t distribution on point *a* and measuring the density of point *b* under that distribution [26]. Points within a circle with a radius of ∼3.5 around each point on the t-SNE map may be considered to be similar to the center point; a group of points that are individually connected by distances within this rough radius can also be considered to be related. (Unlike classical multidimensional scaling, there is no significance assigned to distances larger than 20 on the t-SNE map.) Thus, while 100 clusters were chosen for comparison to *k*-means and other methods (Table 1, Figure 2B), close inspection of the t-SNE map suggested that at least 137 clusters should be partitioned for subsequent analyses from the 2D t-SNE, and 157–167 from 3D t-SNE (Figure S2, Movie S2).

### RCytoscape and Cytoscape

RCytoscape was used to graph networks and manipulate graphs in Cytoscape. RCytoscape (rcytoscape.systemsbiology.net/versions/current/) is a marriage of Cytoscape [44], [45], an open source bioinformatics software platform for visualizing molecular interaction networks, and the broadly popular R language and computing environment for statistical computing and graphics (http://www.r-project.org/), accomplished under the umbrella of Bioconductor (bioconductor.org/), another open source project which provides algorithms and data for bioinformatics in R. The Cytoscape internal Java API is made available through the CytoscapeRPC plugin [64]. Since much of Cytoscape's GUI is built upon that internal java API, RCytoscape is able to present to the R user essentially all the commands on the Cytoscape GUI.

Lung cancer phosphoproteomic data processed as described above was graphed so that node size and increasing yellow color indicates total phosphorylation. RCytoscape setPosition was used to set the position of nodes in Figure S1 using multidimensional scaling coordinates (x–y and x–z) from Spearman, Euclidean, and SED distance matrices, and t-SNE. Subsets of nodes identified as clusters were selected and plotted in a new window, and edges from the edge merge procedure were graphed so that line thickness indicates the overall weight of evidence for interactions between proteins.

### Evaluation of Clusters

The Index used for cluster evaluation is defined as
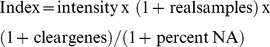
where



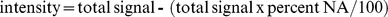


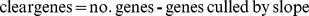


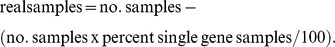



Herein, “single gene samples” is the number of cases where a sample in the cluster contains only one gene, and “single sample genes” represents the number of cases where a gene in the cluster is represented in only one sample. The “culled by slope” function sorts genes and samples from largest to smallest within each cluster and measures the slope of the regression line for each gene in all the samples. If the slope is negative, the gene follows the general pattern in the cluster. If the slope is positive, the gene is more highly expressed in different samples than the rest of the group, and is culled.

The “heatmap2” function from package “gplots” was used to graph data from individual clusters using a blue-yellow color scale, representing NA values as black.

Three dimensional graphs of Pearson and Euclidean distances, and scaled hybrids between the two, and t-SNE embedding were initially explored using R libraries “rgl,” “RGtk2,” “rggobi,” and “scatterplot3d.” The program PyMOL was found to be superior for the purpose of exploring the three-dimensional data structures (Figure S2; Movies S1, S2). Three-dimensional coordinates were scaled to approximate dimensions (in Ångstroms) that PyMOL was designed to handle and exported from R using library “bio3d”. Nodes were represented by small spheres and the data structure. Node identities were preserved in a key. Groups selected in PyMOL were saved as separate PDB files, imported into R using library “bio3d”, and gene names were retrieved using the key.

### External database queries and edge merging

Gene names from clusters identified using the methods above were used to query gene ontology (GO) and protein interaction databases [32]. Gene ontology terms were retrieved using Bioconductor libraries “GO.db,” “GOstats,” and “org.Hs.eg.db” (bioconductor.org/) using a P value <0.01. These data are summarized in GO Summary Tables (Information S1) for the clusters identified by t-SNE on SED embedding. Protein interaction data was retrieved from String (string.embl.de/) [30], GeneMANIA (genemania.org/) [31], and the kinase-substrate data from PhosphoSitePlus (phosphosite.org) [58]. Proteins interaction edges from String included only Experiments and Databases; from GeneMANIA only: Genetic interactions, Pathway, Physical interactions, and Predicted. PhosphoSitePlus edges represent kinase-substrate interactions.

Network graphs that incorporated all the above edges rapidly became too complex to be informative, so edges were merged into a single edge that conveys the total weight of evidence for interaction. Since String and GeneMANIA comprise non-identical but considerably overlapping protein-protein interaction data, we incorporated all edges from each. Quantitative weights from these two databases were summed. We also wished to visualize kinase-substrate relationships from PhosphoSite Plus, and merged these edges, assigning an arbitrary value of 0.25 to PSP edges to ensure that they were visible. Edges were merged (after rechecking ID mapping) assuming that kinase-substrate interactions are directional 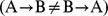 and protein-protein interactions are not 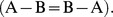


### External Evaluation and Comparison to Random Clusters

To evaluate clusters by external criteria, clusters were compared to two sets of 11 to 35 genes selected at random from the lung cancer dataset. Protein interaction edges from String and GeneMANIA were retrieved as described above except that PhosphoSite edges were not included. This produced a total of 48 random networks, two each with 11 to 35 nodes, iteratively. The number of edges per node was calculated, and their combined weights summed, and divided by the number of nodes. For randomly selected genes from this data set, the average edge weight per node was 0.001931±0.0068, and the average number of edges per node was 0.06473±0.123.

The number of edges expected from a set of nodes (clusternodes) in the entire lung cancer network was calculated using the formula:
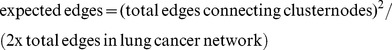



Protein interaction edges from String and GeneMANIA (not PhosphoSite) were used for this calculation. The expected edges was found to be less than the observed edges for network identified by kinases in Figures 3,4,5:



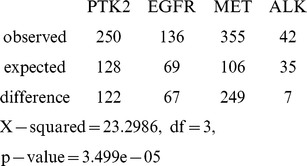



GO terms were retrieved for gene groups determined by clustering methods above, and for the randomly selected genes as described above, using P<0.01. If there is enrichment, at least two genes in the cluster should have the same GO term, so terms with single genes were discarded. Calculations were performed on each set of GO terms to determine the average return of GO terms per gene. The background for randomly selected genes from this data set was 0.35±0.35 GO terms enriched per gene.

## Supporting Information

Figure S1 RCytoscape-driven graphs of lung cancer phosphoproteomic data from Rikova, *et al.,* [24]. RCytoscape makes it possible to set the position of nodes according to multidimensional scaling coordinates derived from statistical measures of relationships among proteins, and to plot different planes of three-dimensional data (*e.g.,* x–y, A, C, E; or x–z, B, D, F). This allows the investigator to zoom in and explore the data using the Cytoscape graphic user interface (GUI). Node size and yellow color intensity indicates greater phosphorylation. Euclidean (A, B) and Spearman (C, D) distances were calculated with NAs in the data set, then remaining NA data were set to 100 times the maximum distance calculated between proteins. Spearman and Euclidean distance matrices were then equally scaled and combined for the Spearman-Euclidean Distance (SED) graph (E, F). Cytoscape does not yet have the ability to plot this data structure in three dimensions, so we used PyMOL to explore the SED data structure using three-dimensional manipulations (Figure S2).
(PDF)

FIgure S2 Spearman and Euclidean distance matrices were combined for the Spearman-Euclidean Distance (SED) graph plotted in three dimensions with PyMOL. Multidimensional scaling was used to determine node coordinates in three dimensions; **A** shows the x–y dimension, **B**, x–z. Groups of proteins (identified by different colors) were selected manually. (**C, D**) Three-dimensional t-SNE embedding of SED dissimilarity plotted as in A and B. 49 groups of proteins (identified by different colors, filtered by low percent NA in the primary data) were selected using a minimum spanning tree method [63] that finds groups that can be connected by a single linkage.
(PDF)

Figure S3 Two-dimensional t-SNE embedding of Spearman-Euclidean dissimilarity graphed in Cytoscape with RCytoscape. Total phosphorylation is represented by node size and color as in Figure S1. Node position was adjusted slightly for clarity.
(PDF)

Figure S4 Heat maps of clusters that contained the most highly tyrosine phosphorylated proteins in lung cancer samples, which ranked at the top based on the index for evaluation (see Materials and Methods). Clusters from (**A**) *k*-means on Euclid dissimilarity; (**B**) t-SNE on on Euclid dissimilarity; (**C**) t-SNE on Spearman dissimilarity; (**D**) t-SNE on Spearman-Euclid dissimilarity; (**E**) filtered combined cluster from (**B**) and (**C** top). In (**C**), the third-ranked cluster containing LCK is also shown (bottom); LCK was included in all the other top-ranked clusters. Data are graphed as a heat map in which black represents NA and increasing scaled peptide counts are shown on a blue-yellow scale (color keys are shown at the left). Data are ordered by decreasing sums of scaled peptide counts for genes (decreasing from top to bottom) and samples (decreasing from left to right).
(JPG)

Figure S5 Heat maps of clusters that ranked second for contents of tyrosine phosphorylated proteins in lung cancer samples, graphed as in Figure S4, derived from (**A**) *k*-means on Euclid dissimilarity; (**B**) t-SNE on on Euclid dissimilarity; (**C**) t-SNE on Spearman dissimilarity; (**D**) t-SNE on Spearman-Euclid dissimilarity; (**E**) filtered combined cluster from (**B**) and (**C**). EGFR was in all of these clusters except that derived from *k*-means on Euclid dissimilarity (**A**), where it was included in the top-ranked cluster (Figure S4A).
(JPG)

Figure S6 Heat maps of clusters that contained MET, graphed as in Figure S4, derived from (**A**) *k*-means on Euclid dissimilarity; (**B**) t-SNE on Euclid dissimilarity (low-abundance data filtered); (**C**) t-SNE on Spearman dissimilarity; and (**D**) t-SNE on Spearman-Euclid dissimilarity.
(JPG)

Figure S7 Heat maps of clusters that contained ALK, graphed as in Figure S4 (color keys omitted), derived from (**A**) *k*-means on Euclid dissimilarity; (**B**) t-SNE on Euclid dissimilarity; (**C**) t-SNE on Spearman dissimilarity; and (**D**) t-SNE on Spearman-Euclid dissimilarity (low-abundance data filtered).
(PDF)

Figure S8 Heat maps of combined filtered clusters that contained ALK and EML4, graphed as in Figure S7. **A**) Combined clusters containing EML4 from t-SNE on Euclidean and Spearman embedding. Samples that did not contain EML4 were filtered. **B**) Combined clusters containing both ALK and EML4 from t-SNE on Euclidean and Spearman embedding. Samples that did not contain ALK or EML4 were filtered. **C**) Combined clusters from t-SNE on SED and Spearman embedding, filtered for samples containing ALK.
(PDF)

Information S1 sed2dGO. Clusters identified by t-SNE on SED embedding and GO term summary for clusters identified by t-SNE on SED embedding.
(ZIP)

Movie S1 Spearman-Euclidean Distance (SED) dissimilarity embedded in three dimensions by classic multidimensional scaling (start of movie) and t-SNE (end of movie), graphed with PyMOL. Selected nodes are labelled.
(M4V)

Movie S2 SED dissimilarity embedded in three dimensions using t-SNE graphed as in Movie S1. 49 groups of proteins (identified by different colors) were selected using a minimum spanning tree method [63], which finds groups that can be connected by a single linkage, filtered by low percent NA in the primary data. The start of the movie shows all proteins not in these groups (green); these were filtered out for the final scenes.
(M4V)

Rcommands S1 LC_PLoS_ONE_Rcommands.R. R script commands for processing phosphoproteomic data.
(R) Click here for additional data file.

Rfunctions S1 LC_PLosONE_Rfunctions.R. R and RCytoscape functions for processing phosphoproteomic data and graphing networks.
(R) Click here for additional data file.
