# Supplementary figures and images for: Wrangling Phosphoproteomic Data to Elucidate Cancer Signaling Pathways
Source: PLoS One. 2013 Jan 3;8(1):e52884. doi: 10.1371/journal.pone.0052884 (PMC3536783; doi:10.1371/journal.pone.0052884)

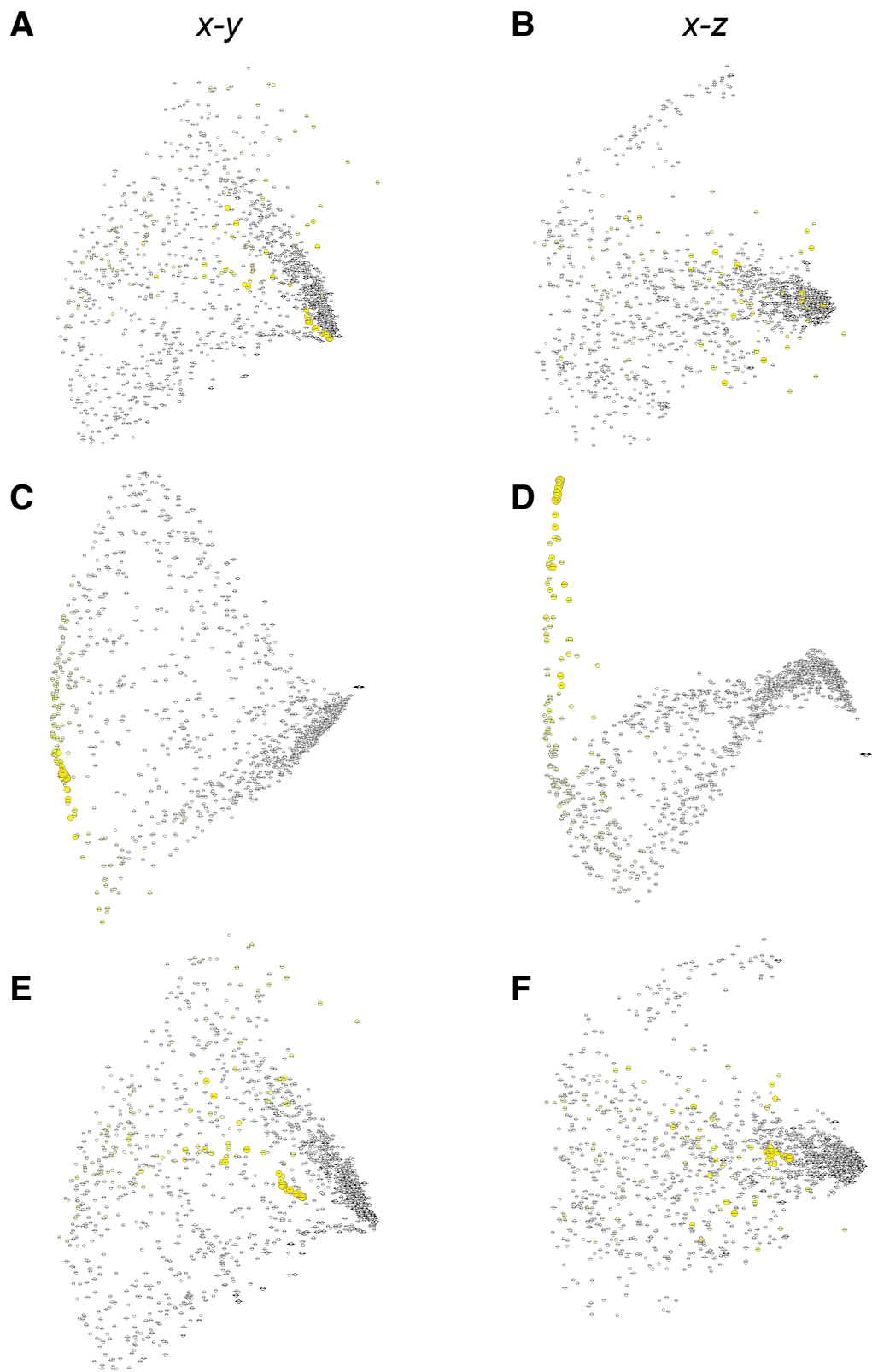

Figure S1

Supplement: Figure S1 — RCytoscape-driven graphs of lung cancer phosphoproteomic data from Rikova, et al., [24]. RCytoscape makes it possible to set the position of nodes according to multidimensional scaling coordinates derived from statistical measures of relationships among proteins, and to plot different planes of three-dimensional data (e.g., x–y, A, C, E; or x–z, B, D, F). This allows the investigator to zoom in and explore the data using the Cytoscape graphic user interface (GUI). Node size and yellow color intensity indicates greater phosphorylation. Euclidean (A, B) and Spearman (C, D) distances were calculated with NAs in the data set, then remaining NA data were set to 100 times the maximum distance calculated between proteins. Spearman and Euclidean distance matrices were then equally scaled and combined for the Spearman-Euclidean Distance (SED) graph (E, F). Cytoscape does not yet have the ability to plot this data structure in three dimensions, so we used PyMOL to explore the SED data structure using three-dimensional manipulations (Figure S2). (PDF) [file pone.0052884.s001.pdf]

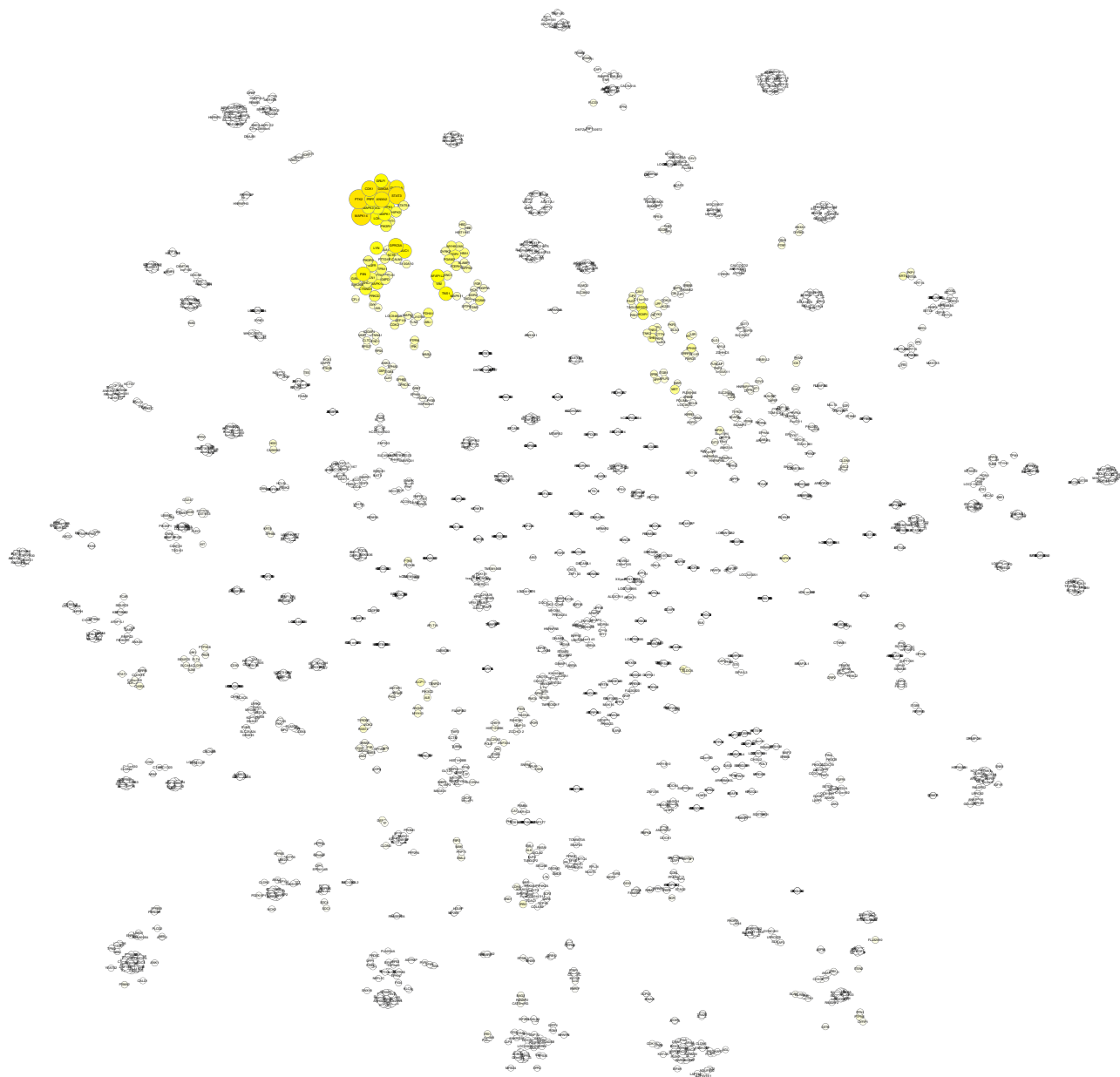

Figure S3

Supplement: Figure S3 — Two-dimensional t-SNE embedding of Spearman-Euclidean dissimilarity graphed in Cytoscape with RCytoscape. Total phosphorylation is represented by node size and color as in Figure S1. Node position was adjusted slightly for clarity. (PDF) [file pone.0052884.s003.pdf]

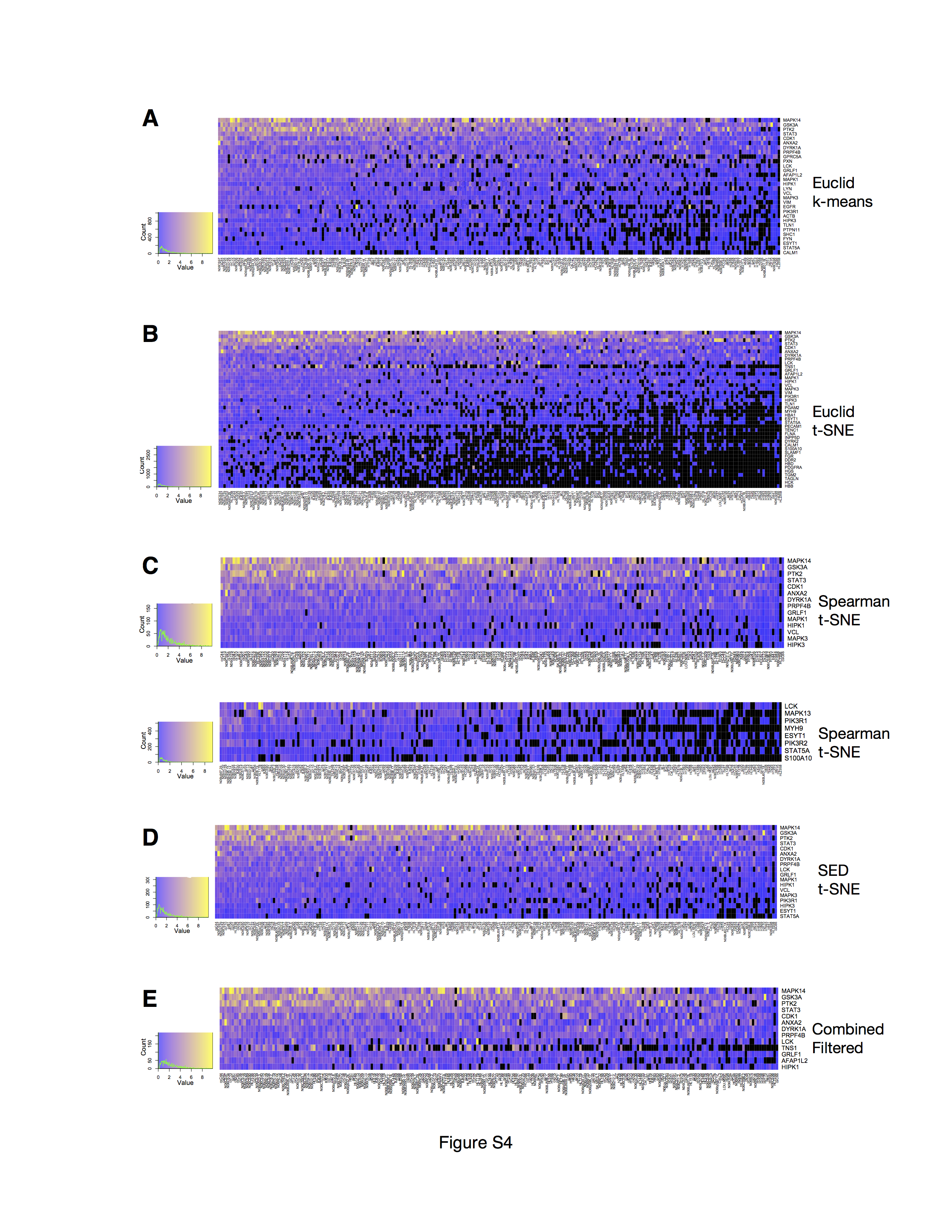

Supplement: Figure S4 — Heat maps of clusters that contained the most highly tyrosine phosphorylated proteins in lung cancer samples, which ranked at the top based on the index for evaluation (see Materials and Methods). Clusters from (A) k-means on Euclid dissimilarity; (B) t-SNE on on Euclid dissimilarity; (C) t-SNE on Spearman dissimilarity; (D) t-SNE on Spearman-Euclid dissimilarity; (E) filtered combined cluster from (B) and (C top). In (C), the third-ranked cluster containing LCK is also shown (bottom); LCK was included in all the other top-ranked clusters. Data are graphed as a heat map in which black represents NA and increasing scaled peptide counts are shown on a blue-yellow scale (color keys are shown at the left). Data are ordered by decreasing sums of scaled peptide counts for genes (decreasing from top to bottom) and samples (decreasing from left to right). (JPG) [file pone.0052884.s004.jpg]

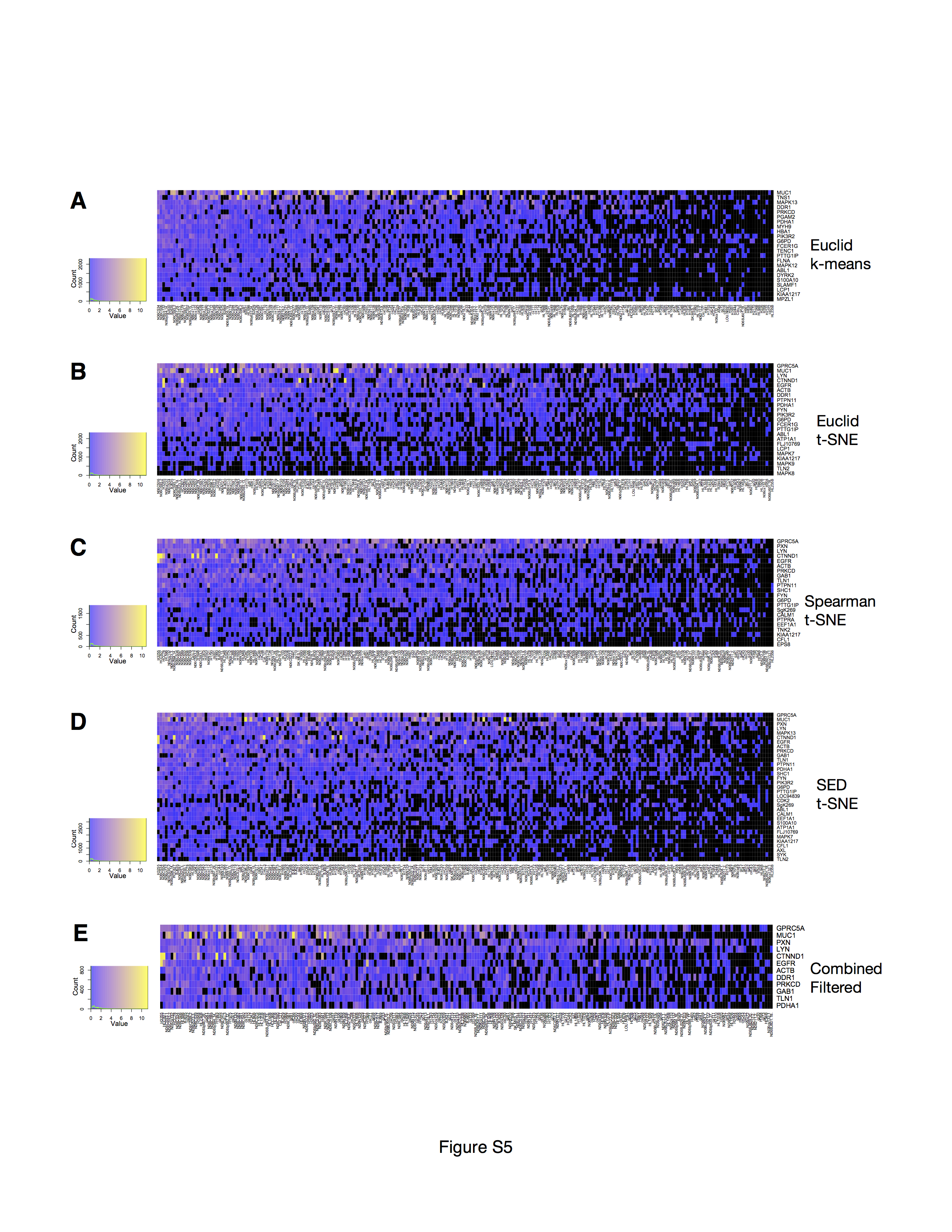

Supplement: Figure S5 — Heat maps of clusters that ranked second for contents of tyrosine phosphorylated proteins in lung cancer samples, graphed as in Figure S4, derived from (A) k-means on Euclid dissimilarity; (B) t-SNE on on Euclid dissimilarity; (C) t-SNE on Spearman dissimilarity; (D) t-SNE on Spearman-Euclid dissimilarity; (E) filtered combined cluster from (B) and (C). EGFR was in all of these clusters except that derived from k-means on Euclid dissimilarity (A), where it was included in the top-ranked cluster (Figure S4A). (JPG) [file pone.0052884.s005.jpg]

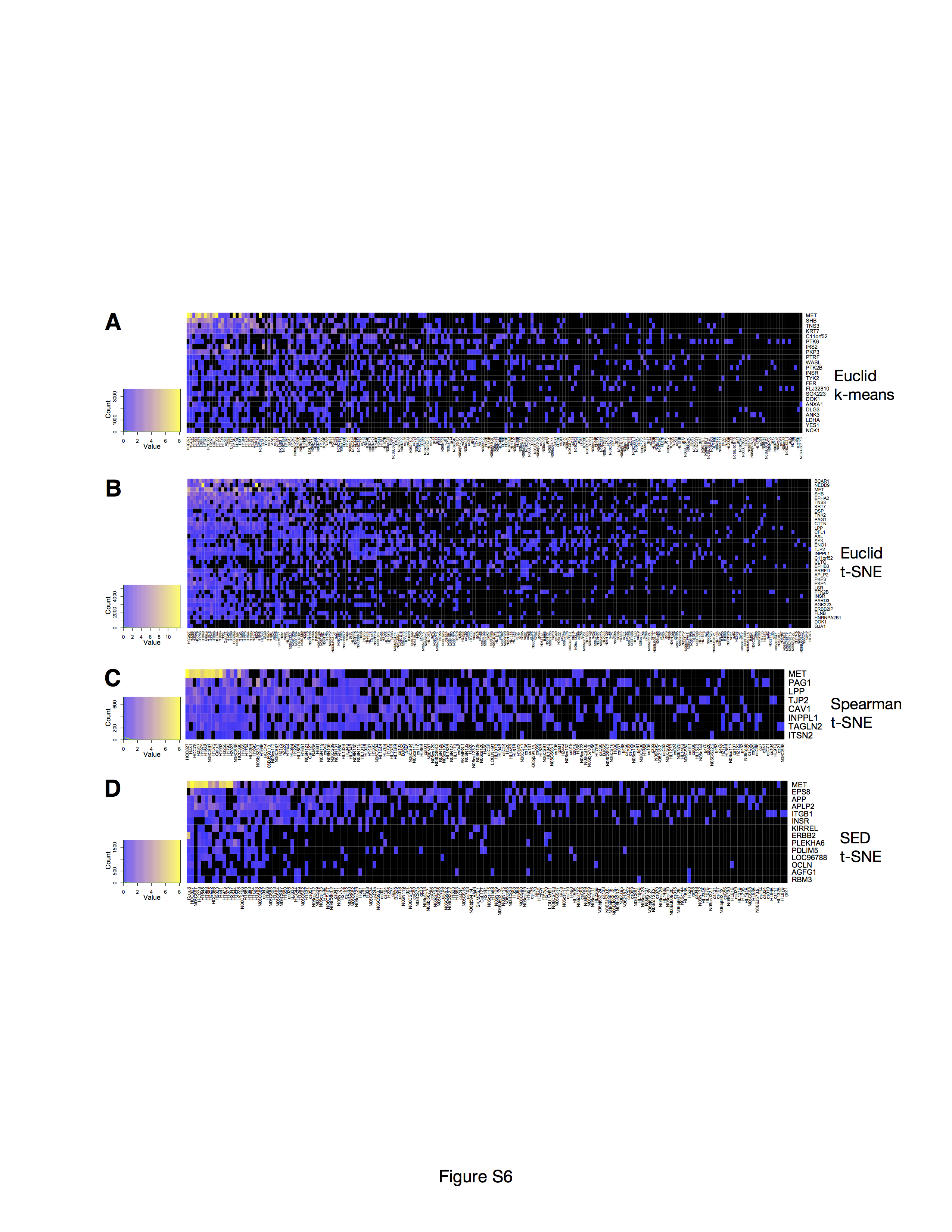

Supplement: Figure S6 — Heat maps of clusters that contained MET, graphed as in Figure S4, derived from (A) k-means on Euclid dissimilarity; (B) t-SNE on Euclid dissimilarity (low-abundance data filtered); (C) t-SNE on Spearman dissimilarity; and (D) t-SNE on Spearman-Euclid dissimilarity. (JPG) [file pone.0052884.s006.jpg]

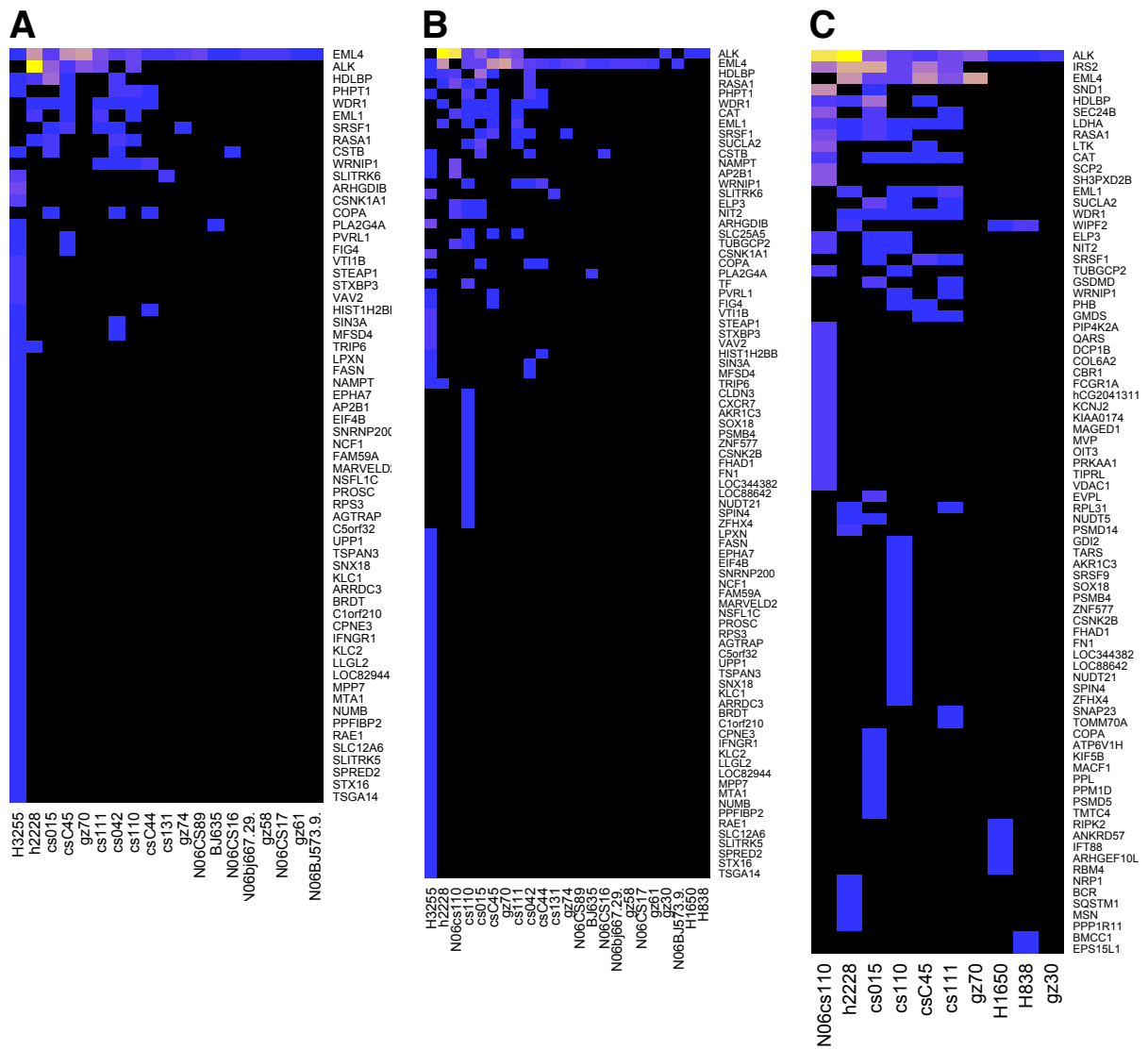

Figure S8

Supplement: Figure S8 — Heat maps of combined filtered clusters that contained ALK and EML4, graphed as in Figure S7. A) Combined clusters containing EML4 from t-SNE on Euclidean and Spearman embedding. Samples that did not contain EML4 were filtered. B) Combined clusters containing both ALK and EML4 from t-SNE on Euclidean and Spearman embedding. Samples that did not contain ALK or EML4 were filtered. C) Combined clusters from t-SNE on SED and Spearman embedding, filtered for samples containing ALK. (PDF) [file pone.0052884.s008.pdf]
