# Supplementary material for: Wrangling Phosphoproteomic Data to Elucidate Cancer Signaling Pathways
Source: PLoS One. 2013 Jan 3;8(1):e52884. doi: 10.1371/journal.pone.0052884 (PMC3536783; doi:10.1371/journal.pone.0052884)
Supplement: FIgure S2 — Spearman and Euclidean distance matrices were combined for the Spearman-Euclidean Distance (SED) graph plotted in three dimensions with PyMOL. Multidimensional scaling was used to determine node coordinates in three dimensions; A shows the x–y dimension, B, x–z. Groups of proteins (identified by different colors) were selected manually. (C, D) Three-dimensional t-SNE embedding of SED dissimilarity plotted as in A and B. 49 groups of proteins (identified by different colors, filtered by low percent NA in the primary data) were selected using a minimum spanning tree method [63] that finds groups that can be connected by a single linkage. (PDF) [file pone.0052884.s002.pdf]

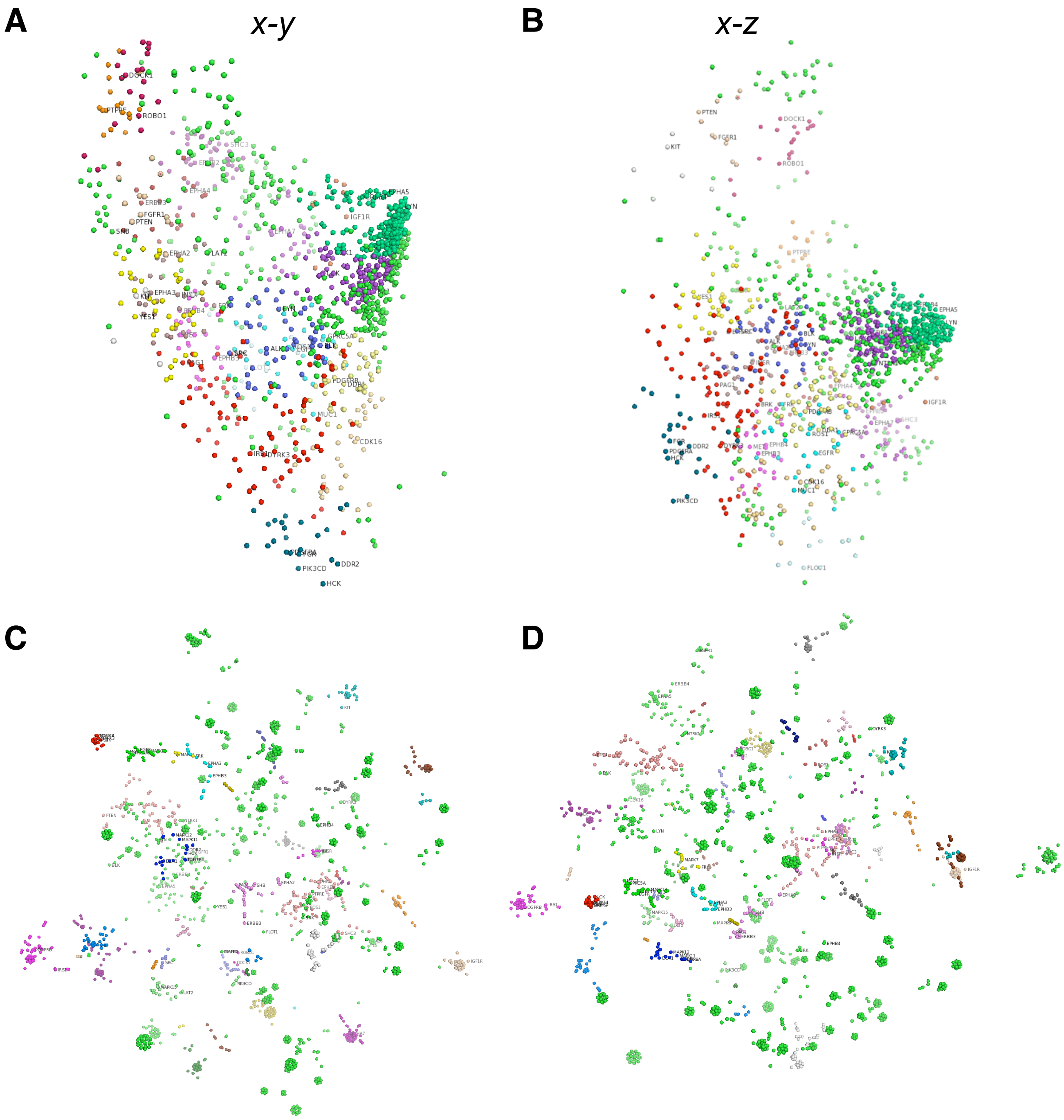

Figure S2
