# Supplementary material for: Wrangling Phosphoproteomic Data to Elucidate Cancer Signaling Pathways
Source: PLoS One. 2013 Jan 3;8(1):e52884. doi: 10.1371/journal.pone.0052884 (PMC3536783; doi:10.1371/journal.pone.0052884)
Supplement: Figure S7 — Heat maps of clusters that contained ALK, graphed as in Figure S4 (color keys omitted), derived from (A) k-means on Euclid dissimilarity; (B) t-SNE on Euclid dissimilarity; (C) t-SNE on Spearman dissimilarity; and (D) t-SNE on Spearman-Euclid dissimilarity (low-abundance data filtered). (PDF) [file pone.0052884.s007.pdf]

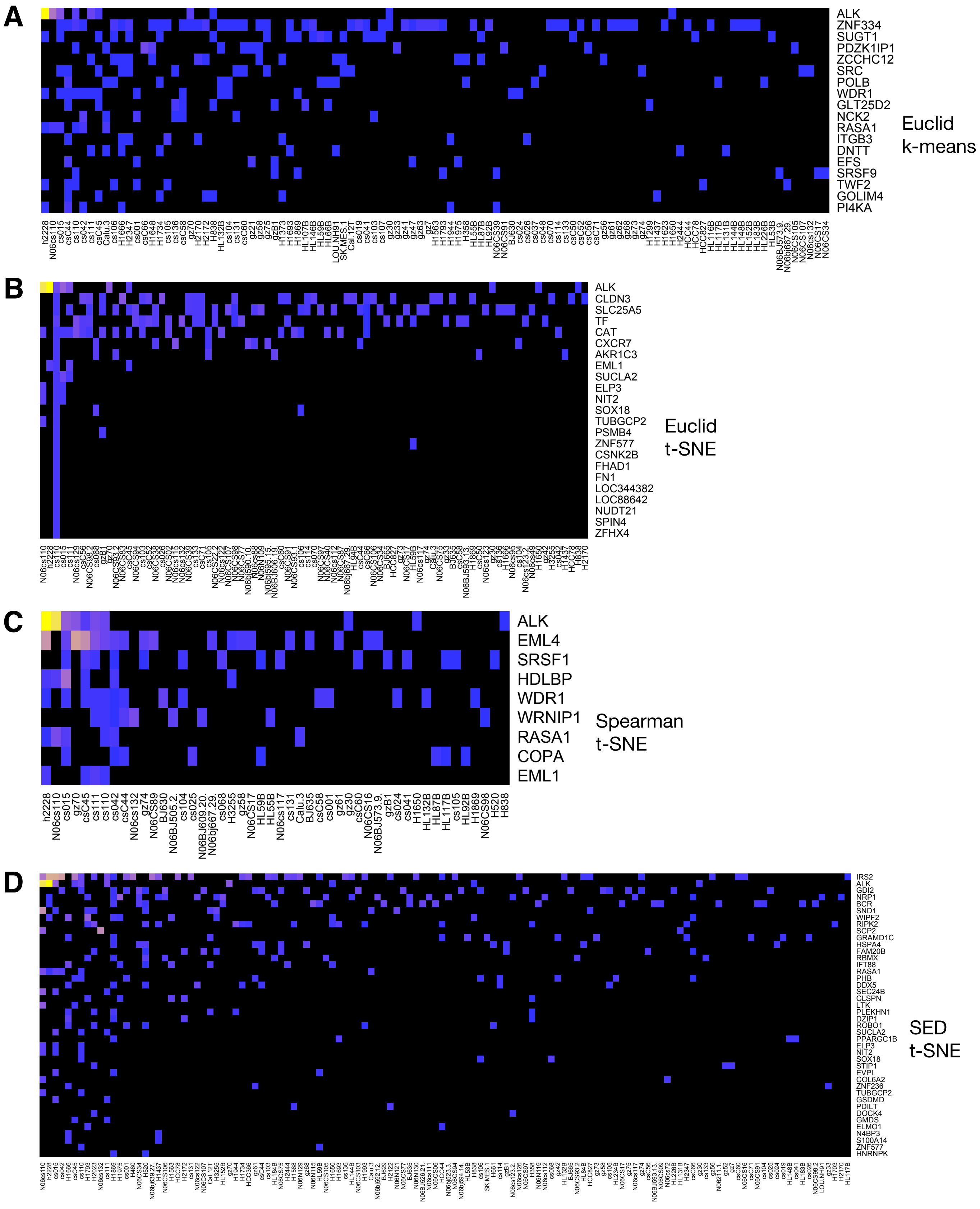
A
ALK
ZNF334
SUGT1
PDZK1IP1
ZCCHC12
SRC
POLB
WDR1
GLT25D2
NCK2
RASA1
ITGB3
DNTT
EFS
SRSF9
TWF2
GOLIM4
PI4KA
Euclid
k-means
B
ALK
CLDN3
SLC25A5
TF
CAT
CXCR7
AKR1C3
EML1
SUCLA2
ELP3
NIT2
SOX18
TUBGCP2
PSMB4
ZNF577
CSNK2B
FHAD1
FN1
LOC344382
LOC88642
NUDT21
SPIN4
ZFHX4
Euclid
t-SNE
C
ALK
EML4
SRSF1
HDLBP
WDR1
WRNIP1
RASA1
COPA
EML1
Spearman
t-SNE
D
IRS2
ALK
GDI2
NRP1
BCR
SND1
WIPF2
RIPK2
SCP2
GRAMD1C
HSPA4
FAM20B
RBMX
IFT88
RASA1
PHB
DDX5
SEC24B
CLSPN
LTK
PLEKHN1
DZIP1
ROBO1
SUCLA2
PPARGC1B
ELP3
NIT2
SOX18
STIP1
EVPL
COL6A2
ZNF236
TUBGCP2
GSDMD
PDILT
DOCK4
GMDS
ELMO1
N4BP3
S100A14
ZNF577
HNRNPK
SED
t-SNE

Figure S7
